# Precision Oncology and Systemic Targeted Therapy in Pseudomyxoma Peritonei

**DOI:** 10.1158/1078-0432.CCR-23-4072

**Published:** 2024-07-11

**Authors:** Jordi Martínez-Quintanilla, Débora Cabot, Doménico Sabia, Oriol Arqués, for the PMPnet Group, Jordi Vergés, Irene Chicote, Lana Bijelic, for the PMPnet Group, Laia Cabellos, Anna M. Alcántara, Isabel Ramos, Pedro Barrios, Oriol Crusellas, Lina M. Palacio, for the PMPnet Group, Juan A. Cámara, Jorge Barriuso, for the PMPnet Group, Juan J. Jiménez, Pau Muñoz-Torres, Lara Nonell, Raquel Flores, Enzo Médico, Marcello Guaglio, for the PMPnet Group, Javier Ros, Elena Élez, Josep Tabernero, Omer Aziz, Marcello Deraco, Héctor G. Palmer, for the PMPnet Group, PMPnet Group, Chiorino Giovanna, Mazzarotto Francesco, Gariboldi Manuela, Varinelli Luca, Cavalleri Tommaso, Chakrabarty Bipasha, Nagaraju Raghavendar, Caswell Patrick, McAllister Milly

**Affiliations:** 1 Translational Program, Stem Cells and Cancer Laboratory, Vall d’Hebron Institute of Oncology (VHIO), Vall d’Hebron Barcelona Hospital Campus, Barcelona, Spain.; 2 Peritoneal Surface Malignancies Surgery Unit, Hospital Sant Joan Despí, Moises Broggi, Sant Joan Despí, Spain.; 3 Department of General Surgery, Hospital Sant Joan Despí, Consorci Sanitari Integral, Sant Joan Despí, Spain.; 4 Former Peritoneal Surface Malignancies Surgery Unit, Hospital Sant Joan Despí, Moises Broggi, Sant Joan Despí, Spain.; 5 Department of General Surgery, Hospital de Barcelona, Assistència Sanitària Col·legial, Barcelona, Spain.; 6 Preclinical Therapeutics Core, University of California, San Francisco, California.; 7 Division of Cancer Sciences, School of Medical Sciences, Faculty of Biology, Medicine and Health, The University of Manchester, Manchester, United Kingdom.; 8 Colorectal and Peritoneal Oncology Centre, The Christie NHSFT, Manchester, United Kingdom.; 9 Preclinical Imaging Platform, Vall d’Hebron Institute of Oncology (VHIO), Barcelona, Spain.; 10 Bioinformatics Unit, Vall d’Hebron Institute of Oncology (VHIO), Barcelona, Spain.; 11 Department of Oncology, University of Turin, Turin, Italy.; 12 Candiolo Cancer Institute, FPO-IRCCS, Candiolo, Italy.; 13 Consultant Surgeon, Peritoneal Surface Malignancies Unit, Division of Colorectal Surgery, National Cancer Institute, Milan, Italy.; 14 Medical Oncology Service, Vall d’Hebron Institute of Oncology (VHIO), Vall d’Hebron Barcelona Hospital Campus, Barcelona, Spain.; 15 CIBERONC, Madrid, Spain.; 16 Peritoneal Surfaces Malignance Unit, Fondazione IRCCS Instituto Nazionale dei Tumori, Milan, Italy.

## Abstract

**Purpose:**

Pseudomyxoma peritonei (PMP) is a rare and poorly understood malignant condition characterized by the accumulation of intra-abdominal mucin produced from peritoneal metastases. Currently, cytoreductive surgery remains the mainstay of treatment but disease recurrence and death after relapse frequently occur in patients with PMP. New therapeutic strategies are therefore urgently needed for these patients.

**Experimental Design:**

A total of 120 PMP samples from 50 patients were processed to generate a collection of 50 patient-derived organoid (PDO) and xenograft (PDX) models. Whole exome sequencing, immunohistochemistry analyses, and *in vitro* and *in vivo* drug efficacy studies were performed.

**Results:**

In this study, we have generated a collection of PMP preclinical models and identified druggable targets, including *BRAF*^V600E^, *KRAS*^G12C^, and *KRAS*^G12D^, that could also be detected in intra-abdominal mucin biopsies of patients with PMP using droplet digital PCR. Preclinical models preserved the histopathological markers from the original patient sample. The BRAF^V600E^ inhibitor encorafenib reduced cell viability of *BRAF*^V600E^ PMP-PDO models. Proof-of-concept *in vivo* experiments showed that a systemic treatment with encorafenib significantly reduced tumor growth and prolonged survival in subcutaneous and orthotopic *BRAF*^V600E^-PMP-PDX mouse models.

**Conclusions:**

Our study demonstrates for the first time that systemic targeted therapies can effectively control PMP tumors. BRAF signaling pathway inhibition represents a new therapeutic opportunity for patients with *BRAF*^V600E^ PMP who have a poor prognosis. Importantly, our present data and collection of preclinical models pave the way for evaluating the efficacy of other systemic targeted therapies toward extending the promise of precision oncology to patients with PMP.

**Translational Relevance**

Currently, cytoreductive surgery remains the only therapeutic option available for patients with Pseudomyxoma peritonei (PMP). Therefore, new therapeutic strategies are urgently needed for these patients. We present a unique collection of patient-derived organoids (PDO) and xenografts (PDX) from patients with PMP and showed that they are reliable preclinical models to study this disease. Genomic characterization of these models revealed *KRAS* and *BRAF* druggable targets. Moreover, droplet digital PCR showed to be a useful diagnostic tool to detect these druggable mutations in the intra-abdominal mucin biopsies of patients with PMP. In a proof-of-concept study, *BRAF*^V600E^ PDO and PDX preclinical models responded to systemic targeted therapy. In conclusion, our study paves the way for evaluating systemic targeted therapies for patients with PMP in the clinic.

## Introduction

Pseudomyxoma peritonei (PMP) is a rare malignant condition that usually begins in the appendix with an incidence of one to three cases per million per year (1). It is characterized by the progressive accumulation of intra-abdominal mucin and the development of peritoneal metastases (PM) arising mostly from appendiceal mucinous neoplasms (AMN) but also ovarian, small bowel, or colorectal cancer. Peritoneal Surface Oncology Group International (PSOGI) pathological classification system distinguishes between characteristics associated with the primary AMN and those linked to the PM (2), as these metastases are the underlying cause of the clinical syndrome known as pseudomyxoma. Specifically, PM are categorized into acellular mucin, low-grade mucinous carcinoma peritonei (G1), high-grade mucinous carcinoma peritonei (G2), and high-grade mucinous carcinoma peritonei (G3) with the presence or absence of signet ring cells (SRC). Primary appendiceal tumors can be classified as low-grade AMN (LAMN), high-grade AMN (HAMN), and infiltrative appendiceal adenocarcinoma that can be graded as G1, G2, and G3.

The most effective loco-regional treatment currently employed in these patients involves cytoreductive surgery (CRS) and hyperthermic intraperitoneal chemotherapy (HIPEC) based on platin salts (cisplatin or oxaliplatin) or mitomycin C. However, patients with PMP develop early recurrence and ultimately succumb to intra-abdominal disease progression (3–5). Preclinical studies of systemic treatment regimens have not shown efficacy (6). Indeed, therapeutic efficacy of systemic chemotherapy protocols in patients with PMP has not been validated yet because large, randomized trials have not been performed. Therefore, there is an urgent medical need to provide new therapeutic strategies for these patients.

Little is known about the molecular events underlying the origin and progression of PMP. Performing whole exome sequencing (WES) on a significant cohort of patients with PMP has been a real challenge in the past due to the low incidence of this malignant condition and the very limited amount of tumor cells (often less than 5% of cellularity; ref. 7). Despite these difficulties, previous WES studies in paraffin sections of tumors from patients with PMP have shown recurrent mutations in *KRAS* and *GNAS* (8). Regarding *KRAS* mutations, previous studies have mainly shown three different missense alterations G12D, G12V, and A146T. Other mutated genes have been occasionally identified including *AKT1*, *FAT4*, *TGFBR1*, *TP53*, *SMAD3/4*, *ATM*, and *BRAF* (9, 10). *BRAF* mutations, while rare in patients with PMP (9, 10), have been associated with high-grade cases (11) that typically show the worst prognosis (12).

The lack of relevant preclinical models has hampered proof-of-concept studies to test matched targeted therapies and extend the promise of precision oncology to patients with PMP (13). Over the past decade, three-dimensional (3D) patient-derived organoid (PDO) cultures and *in vivo* patient-derived xenograft (PDX) animal models have enabled the expansion of primary tumor cells in the laboratory. These preclinical models have shown to faithfully recapitulate patient tumor biology and response to anticancer drugs (14, 15). Recent studies have established PDO or PDX organoid (PDXO) models derived from appendiceal tumor samples (6, 16) and they have shown to predict response to chemotherapy and immunotherapy (6, 17). However, no organoid model derived from samples of patients with PMP has been established up to date. These preclinical models could greatly facilitate deep sequencing without the limitations of low cellularity present in tumor samples from patients with PMP as well as drug screenings (14, 15).

Several groups have established orthotopic PMP-PDX models and shown that they preserved the histologic features of the original sample from patients with PMP, such as histological markers and abundant mucinous tissue (18). PMP-PDX models have been successfully used to evaluate the efficacy of intraperitoneal chemotherapeutic regimens (6, 19). Preclinical studies with a *BRAF* mutant PMP-PDX model showed resistance to different chemotherapy drugs compared with *KRAS* mutant PMP-PDX models (19), suggesting that *BRAF* mutation could be associated with a poor prognosis.

The lack of investment and appropriate resources for investigating rare diseases also frequently hinders the development of effective drugs, and the low number of eligible trial participants complicates and delays clinical studies. To help overcome these challenges, an impactful and rapid strategy could be to extend the evaluation of therapies already approved by the FDA for prevalent cancers to rare diseases such as PMP.

Over the last decade, the development of targeted therapies has revolutionized the oncology field and provided many patients with new opportunities (20). In this line, BRAF inhibitors are small-molecule drugs that bind and inactivate the mutated *BRAF*^V600E^. We previously demonstrated the therapeutic efficacy of encorafenib (BRAF inhibitor) in combination with the epidermal growth factor receptor (EGFR) inhibitor cetuximab for the treatment of patients with advanced colorectal cancer (21). These excellent results in the BEACON trial (21) led to a recently approved standard-of-care for patients with *BRAF*^V600E^ mutant colorectal cancer who have previously progressed to at least one previous chemotherapy regimen. Interestingly, in this and previous studies, colorectal cancer peritoneal metastases are more prevalent in patients with *BRAF*^V600E^ mutant tumors (21), suggesting that the abnormal activation of this oncogene could particularly promote the growth of peritoneal tumors of different kinds including aggressive PMP.

In this study, we generated a collection of PMP-organoid models and identified druggable targets by WES. Droplet digital PCR (ddPCR) from mucin biopsies from our patients with PMP was used to detect druggable mutations as a molecular screening strategy to offer tailored systemic therapy. As a proof-of-concept, we demonstrated therapeutic efficacy of the BRAF^V600E^ inhibitor encorafenib in PMP preclinical models. Importantly, our results pave the way for investigating novel systemic targeted therapies and validating them in our collection of preclinical models, toward extending the promise of precision oncology to patients with PMP with new systemic treatment opportunities.

## Materials and Methods

### Patient samples

A total of 212 fresh tumor tissue samples were acquired from 50 patients with PMP arising from appendix (47 patients), ovarian (two patients), and unknown (one patient) origin undergoing CRS at the Hospital Sant Juan Despí Moisès Broggi (San Juan Despí, Spain) or Hospital de Barcelona (Barcelona, Spain). Following PSOGI classification (2), primary tumor samples were classified as: LAMN (15 samples), LAMN/HAMN (one sample), or HAMN (five samples). Infiltrative appendiceal adenocarcinoma was not included in this study. PM were classified as: acellular mucin (30 samples), low-grade (G1) mucinous carcinoma peritonei (124 samples), high-grade (G2) mucinous carcinoma peritonei (33 samples), or high-grade (G3) mucinous carcinoma peritonei with or without SRC (four samples).

Patient 5 was a 69-year-old woman who was referred to the Hospital de Barcelona due to abdominal distension and was diagnosed with a high-grade (G3) mucinous carcinoma peritonei and a Sugarbaker peritoneal carcinomatosis index (PCI) of 32/39. The extension and the aggressiveness of the tumor mass, that most probably destroyed the appendix, made it difficult to identify the appendiceal primary tumor. However, a right hemicolectomy was performed and no tumor cells were detected in the colon of the patient. Therefore, the malignant condition of this patient was classified as PMP of unknown origin although most probably of an appendiceal origin. During surgery, disseminated peritoneal implants, omentum, right diaphragm implants, pelvis implants, and Glisson capsule were removed and some of these tissues were collected for processing in the laboratory. After complete cytoreduction, HIPEC with intraperitoneal oxaliplatin was administered for 30 minutes at 42.5°C.

The studies were conducted in accordance with the Declaration of Helsinki ethical guidelines. Written informed consent was obtained from all patients and the project was approved by the Research Ethics Committee of the Hospital Universitari of Bellvitge, Barcelona, Spain (approval ID: PR315/21).

### Histological classification of samples from patients with PMP

Hematoxylin & eosin (H&E) staining from all the 212 samples from patients with PMP was performed in the pathology department at Vall d’Hebron University Hospital following routine protocols. H&E staining was analyzed by two independent pathologists from different hospitals in Sant Joan Despí and Barcelona, Spain (Moises Broggi Hospital and Vall d’Hebron University Hospital) to histologically classify the PMP samples following PSOGI guidelines from 2016 (2) and to determine the percentage of neoplastic cellularity in each sample.

### Processed samples and organoid cultures

Fresh tumor samples were processed within 2 hours after surgery by dissociating the tissue in small fragments. Enzymatic digestion was performed using collagenase (1.5 mg/mL; Sigma-Aldrich, C0130) and DNase I (20 μg/mL; Sigma-Aldrich, D4263-5VL) in a medium [Dulbecco’s Modified Eagle Medium (DMEM)/F12 (Gibco, 21331-020)] supplemented with a cocktail of antibiotics and antifungals [penicillin/streptomycin (100 units/mL, 100 μg/mL; Sigma. 15140-122), fungizone (10 μg/mL; Gibco, 15290-026), kanamycin (10 μg/mL; Gibco, 15160-047), gentamycin (50 μg/mL; Gibco, 15750-037), and nystatin (5 μg/mL; Sigma-Aldrich, N4014)] during 30 minutes at 37°C with intermittent pipetting every 10 minutes to dissociate cells (22). After the incubation a volume of basal media [Advanced DMEM/F12 (Gibco, 12634-010)] supplemented with penicillin/streptomycin (100 units/mL, 100 μg/mL), Hepes [10 mmol/L; Sigma, H0887, RRID:SCR_000488) and Glutamax (1/100; Gibco, 35050-038)] was added, and the dissociated sample was then filtered (100 μm pore size; BD Biosciences, 352360) and washed with fresh basal media. Cellular pellet was mixed with Matrigel (Corning, 356255) and plated in six-well plates in drops of 25 μL. The plate was placed in the incubator at 37°C for 10 to 15 minutes and 2 to 3 mL Human Intestinal Stem Cell Media (HISC adapted from ref. 23: 30% Wnt3A conditioned medium + 15% RSPO1 conditioned medium + 7,5% noggin conditioned medium + Basal culture medium) supplemented with B27 (1X; Gibco, 17504044), N2 (1X; Gibco, 17502048), N-acetyl-L-cysteine (1,35 mmol/L; Sigma-Aldrich, A9165-5G), nicotinamide (5 mmol/L; Sigma-Aldrich, N3376), human EGF (50 ng/mL; PrepoTech, AF-100-15), [Leu^15^]-Gastrin I (10 nmol/L; Sigma-Aldrich, G9145-1mg), prostaglandin E2 (10 nmol/L; Sigma-Aldrich, P0409), A83-01 (500 nmol/L; Tocris, 2939), SB202190 (3 μmol/L; Sigma-Aldrich, S7067) and Normocin (125 μg/ml; Invivogen, ant-nr-2) was added and replaced every 2 to 3 days. Conditioned media were obtained from confluent L1wnt3A cell line (Wnt3A conditioned medium), 293t RSPO-mCherry cell line (RSPO1 conditioned medium), and 293t Noggin-mCherry cell line (noggin conditioned medium) cultured in basal media.

When organoid cultures were confluent, media was aspirated, and the ice-cold Cell Recovery Solution (Corning, 354253) was added, and the plate incubated at 4°C for 30 minutes following the manufacturer’s protocol. Organoids were collected, washed with basal media, dissociated by pipetting up and down, and expanded by seeding them with Matrigel. Organoid growth was monitored weekly for the detection of initiated organoids, and organoids were kept in the same passage for no longer than 2 weeks. The number of passages between the generation of the PDO model and the use in the experiments described below was less than five. Mycoplasma testing was not conducted in PDO models.

For *in vitro* experiments with drugs, TrypLE Express (Gibco, 12605010) was used to obtain a single-cell suspension. Organoids were mixed with 200 to 400 μL TrypLE and incubated at 37°C for 2 to 3 minutes, then mixed up and down by pipetting, washed with basal media, and counted using a Neubauer chamber. After cell dissociation, plated organoids were cultured with HISC media complemented with Rhock (10 μmol/L; Y-27632, CalbioChem9) and GSK3 (5 μmol/L; SML1046, Sigma-Aldrich) inhibitors.

### Colorectal cancer PDXO models

Colorectal cancer PDXO model T70 (*KRAS*^WT^ and *BRAF*^WT^), T148 (*KRAS*^WT^ and *BRAF*^WT^), CTAX34 (*KRAS*^WT^ and *BRAF*^V600E^), and CTAX26 (*KRAS*^G12D^ and *BRAF*^WT^) were derived from a collection of colorectal cancer PDX from Héctor G. Palmer’s laboratory (24). Briefly, subcutaneous colorectal cancer PDX tumors were dissociated with collagenase and DNase I for 30 minutes at 37°C, as previously mentioned. Cellular pellet was mixed with Matrigel and plated in six-well plates with HISC media complemented with Rhock and GSK3 inhibitor to generate PDXO cultures. PDXO models were kept in the same passage for no longer than 2 weeks. The number of passages between thawing and use in the experiments described below was less than five. Mycoplasma testing was not conducted in PDXO models.

### *In vitro* targeted therapy experiments

Single-cell organoid suspension from PMP-PDO (PMP5.2), PMP-PDXO (PMP5.1 or PMP53.1), CRC-PDXO (T70, T148, CTXA26 or CTAX34), or melanoma cell line A375 (RRID:CVCL_0132) from the ATCC were used for *in vitro* experiments. The number of passages between thawing and use in the experiments described below was less than five. Mycoplasma testing was not conducted. 1.5 × 10^3^ to 5 × 10^3^ cells were plated in a 5 μL drop of Matrigel (cells diluted in basal media and mixed with Matrigel in a proportion 1:4) in a 96-well plate and cultured with HISC media for 24 hours. The next day, organoids were cultured under serum-limiting condition (DMEM 0.5% FBS or HISC media without EGF). After overnight, cells were treated with mitomycin C (150 ng/mL; Sigma, M0503), cetuximab (100 μg/mL; MERCK, 658752), encorafenib (1 μmol/L; MedChemExpress, HY-15605), Binimetinib (200 nmol/L; MedChemExpress, HY-15202), MRTX1133 (HY-134813, MedChermExpress), oxaliplatin (O9512-5mg, Sigma-Aldrich), or vehicle in DMEM 2.5% FBS or HISC media. At 48 hours, organoids were retreated with the corresponding drug. At 5 days or 48 hours after the initial treatment, CellTiter-Glo (Promega, G7571) or Caspase-Glo 3/7 3D Assay (Promega, G8983) were used (following the manufacturer’s protocol) to determine cell viability or cell apoptosis, respectively.

### Paraffin-embedded PDOs

PDOs were removed from Matrigel using Cell Recovery Solution at 4°C for 30 minutes, rinsed three times with cold PBS, and centrifuged at 830 g for 5 minutes without disturbing the pellet. The resulting PDO pellet was fixed with 1 mL 4% paraformaldehyde (Santa Cruz biotechnology, sc-281692) at 4°C overnight. The fixing solution was then replaced with 70% ethanol and PDO pellet was dehydrated and embedded in paraffin blocks. Finally, paraffin-embedded PDO sections were cut 4-μm thick and analyzed by immunohistochemistry (IHC) with specific antibodies (Supplementary Table S1).

### Generation of PDX models

Animal experiments were conducted following the European Union’s animal care directive (86/609/EEC) and were approved by the Ethical Committee of Animal Experimentation of the Vall d’Hebron Research Institute—VHIR (ID 12/18 and 38/23 CEEA). Subcutaneous PMP-PDX models were generated by mixing 2 × 10^5^ PDO cells (PMP5.1 or PMP5.4) resuspended in 50 μL of PBS with 50 μL of Matrigel and injecting them subcutaneously into a flank of a NOD-SCID (NOD.CB17-Prkdc^scid^*/*NcrCrl) mouse (Charles River and Janvier Laboratories). Tumors were measured every 2 to 3 days with a caliper until they reached 1 cm^3^, when animals were euthanized, and the tumors removed.

To generate orthotopic PMP models, fresh PMP tumor samples were cut into 0.5 cm^3^ fragments and two to four pieces were surgically implanted in the peritoneal cavity of NOD-SCID mice, as previously described (18, 25). Animals were monitored two times a week and once an increase in peritoneal volume was observed mice were euthanized and PMP-PDX tumor isolated. In the instance that the tumor was a mucinous suspension, 150 to 500 μL was reimplanted in a new mouse by intraperitoneal injection using a 21G needle. When the tumor was semi-solid it was cut and expanded in a new mouse by intraperitoneal surgery as explained above.

### Immunohistochemical stain

Formalin-fixed, paraffin-embedded (FFPE) samples from patients with PMP, PDO pellets, or PDX tissue sections were routine deparaffinated, rehydrated, and treated with 1 mmol/L EDTA, 10 mmol/L Tris Base, and 0.05% Tween-20 pH 9 buffer for CDX2 or with 10 mmol/L sodium citrate pH 6 buffer for Cleaved-Caspase 3, Ki-67, and MUC2.

After blocking endogenous peroxidase activity, slides were permeabilized with 1% Tween-20, except for CDX2 that was in 1% Triton in PBS for 15 minutes. Tissue specimens were then blocked for 1 hour with 3% BSA in PBS and incubated with corresponding primary antibodies (Supplementary Table S1) diluted in blocking solution at 4°C overnight. For chromogenic detection, after washing, sections were incubated with corresponding HRP-conjugated secondary antibodies (Supplementary Table S1) at corresponding dilutions for 1 hour at RT. After washing, Chromogen DAB/substrate reagent (DAKO, K3468) was added onto the slides and incubated up to 10 minutes. Finally, the slides were counterstained with hematoxylin, dehydrated, and mounted with DPX (Sigma, 06522).

CK20 and CK7 immunostaining and Alcian blue and H&E staining was performed in the pathology department at our hospital (Vall d’Hebron University Hospital) following routine protocols. NanoZoomer 2.0-HT Digital slide scanner C9600 (Hamamatsu Photonics K.K.) was used to visualize and assess the immunostainings.

### Western blot analysis

Western blots were performed according to standard procedures. Briefly, six-well plate organoid samples were collected and lysed in a buffer containing 50 mmol/L Tris-HCl pH 8.8, 10 mmol/L EDTA pH 8, 1% sodium dodecyl sulfate (SDS), and 1 mmol/L dithiothreitol (DTT) supplemented with protease and phosphatase inhibitor cocktail (Roche, 53875100 and 04906837001). Then, samples were boiled at 95°C in a dry bath for 15 minutes, sonicated, and centrifuged at 10,000 *g* for 1 minute at RT. After that, the supernatant was saved for further protein quantification. Lysates were separated by SDS-PAGE and protein was transferred onto a nitrocellulose membrane (Bio-Rad 1620115). Membranes were blocked with TBS 0.1% Tween-20, and 5% non-fat milk or Bovine Serum Albumin (BSA; Sigma-Aldrich, A3059), incubated with corresponding primary and secondary antibodies (Supplementary Table S1) diluted in blocking solution, and visualized using SuperSignal West Pico Chemiluminescent Substrate (ThermoFisher Scientific, 34580) and IBRIGHT CL 1500 (Invitrogen).

### Genomic analyses

Genomic DNA (gDNA) from normal tissue, PMP-PDO or PMP-PDXO cell cultures were isolated using the QIAamp DNA blood Mini Kit (Qiagen, 51104) and QIAamp DNA Mini Kit (Qiagen, 51304), respectively. gDNA from patient sample 5.1 was isolated from paraffin sections using Maxwell 16 FFPE Plus LEV DNA Purification Kit (Promega, AS1135).

WES was performed by Macrogen, Korea (RRID:SCR_014454), on 200 ng of genomic DNA from healthy tissue, PMP-PDO or PMP-PDXO using TwistCore exome (33 Mb) library prep kit. Each resulting library was sequenced using Novaseq 6000 for 2 × 150 pair end and 4Gb (100×) and 12Gb (300×) for healthy or PMP-PDO samples, respectively.

The resulting reads were processed using Sarek’s pipeline from nf-core project (26) with genome GRCh38. The alignment was performed using the BWA (ref. 27; RRID:SCR_010910) and the calling was performed using Mutect2 (https://doi.org/10.1101/861054). Finally, somatic variants were annotated using SnpEff (ref. 28; RRID:SCR_005191). The most relevant variants were extracted using in-house scripts.

Variant allele frequency (VAF) from a specific mutation was calculated dividing the mutated reads by the total reads of a specific allele. We kept the variants that had a VAF ≥ 5% and those where the tumor sample presented ≥50 reads. When comparing sample 1 from patient 5 against its PDXO model, all variants with a VAF ≥ 5% in the PDXO and present also in the patient sample, regardless of their VAF, were used for the analysis.

Annotated results were manually converted to Mutation Annotation Format (MAF) and results were summarized in an oncoplot using the R packages maftools (29) for reading the data and ComplexHeatmap (30, 31) for visualization. Data for the additional annotations in the oncoplot were obtained from cBioportal [Colorectal Adenocarcinoma: The Cancer Genome Atlas (TCGA), PanCancer Atlas], Ang and colleagues (32), and Alakus and colleagues (33). Mutations in the codons 12/13/146 and codon 600 from the *KRAS* and *BRAF* genes, respectively, were validated by PCR using the following oligos (Supplementary Table S2) and Sanger sequencing (Macrogen Inc).

### Droplet digital PCR

gDNA from tumor samples and intra-abdominal mucin biopsies from PMP patients was isolated from frozen tissue using QIAamp DNA Mini Kit (Qiagen, 51304). The QX200 ddPCR System (Bio-Rad Laboratories, Hercules, CA, RRID:SCR_008426) was used to detect the *KRAS* G12C, *KRAS* G12D and *BRAF* V600E variants. Certified probes of the manufacturer were used for the assays (Bio-Rad). Briefly, the 20 μL final volume of TaqMan PCR reaction mixture was assembled with 1× ddPCR Supermix for Probes (no dUTP; Bio-Rad, 1863023), 900 nmol/L of each primer, 250 nmol/L of each probe (FAM for the mutant and HEX for the WT), and 40  ng of gDNA from patient samples. Each assay was performed in duplicate in separate mixes and loaded in different wells for amplification. The 20 μL reaction mixture was transferred to DG8 cartridges (Bio-Rad, 1864008) and applied to the QX200 Droplet Generator Cartridge (Bio-Rad Laboratories, Hercules, CA) with 70 μL of mineral oil (Bio-Rad, 1864005) to form droplets in ∼40 μL of oil-in-water mixture. The mixture was transferred to a 96-well PCR plate and heat sealed. The plate was placed in a C1000 Touch thermal cycler (Bio-Rad Laboratories) and amplified to the endpoint PCR. Thermal cycling program was performed at 95°C × 10 minutes (1 cycle), 94°C for 30 seconds (1 cycle), 56°C × 1 minute (39 cycles), and 98°C × 10 minutes (1 cycle), with a ramp rate of 2°C per second. After PCR, the 96-well PCR plate was read on a QX-200 Droplet Reader (Bio-Rad Laboratories, RRID:SCR_008426) and the data were analyzed with QuantaSoft version 1.7.4 (RRID:SCR_025321). Known genomic DNA from different PDX tumor models were run as positive or negative controls and used to determine the cutoff for allele calling in each assay. VAF from a specific mutation was calculated dividing the mutated reads by the total reads of a specific allele.

### *In vivo* targeted therapy experiments

Subcutaneous studies were performed using single-cell suspension derived from previously implanted PMP subcutaneous tumors (PMP5.1). Tumors were dissociated as previously published (22) and 1 × 10^5^ tumor cells were resuspended in 50 μL of PBS, mixed with 50 μL of Matrigel, and injected subcutaneously into both flanks of 35 NOD-SCID mice. When tumors reached an average 150 mm^3^, mice were randomized and treated with either: (i) vehicle once per day by oral gavage, (ii) cetuximab (20 mg/kg) twice per week by intraperitoneal injection, (iii) encorafenib (20 mg/kg) once per day by oral gavage, or (iv) doublet (encorafenib and cetuximab). Tumor growth was monitored by caliper measurement three times per week and volume was estimated using the following formula: *V* = (length × width^2^)/2, where length represents the largest tumor diameter and width represents the perpendicular tumor diameter. Animals were euthanized after 44 days of treatment and xenograft tumors were then removed and fixed in paraformaldehyde for histological analysis.

Orthotopic studies were performed using PMP-PDX (PMP5.1) intraperitoneally implanted in NOD-SCID mice. When matching endpoint criteria, animals were euthanized, and mucinous tumor tissue was isolated. A total of 150 μL of PMP-PDX mucinous tissue was mixed with PBS (1:1 volume) and injected intraperitoneally in NOD-SCID mice using a 21G needle. Treatments were initiated 6 days after mucinous tumor administration to simulate the clinical scenario of residual disease after CRS. Animals were randomized and treated with either: (i) vehicle once per day by oral gavage, (ii) cetuximab (20 mg/kg) twice per week by intraperitoneal injection and vehicle once per day by oral gavage, (iii) encorafenib (20 mg/kg) once per day by oral gavage, or (iv) doublet. Animal weight was measured three times per week and endpoint criterion was considered when mice surpassed weight gain of 50%. When matching endpoint criterion mice were euthanized and PDX tumors removed, quantified (mL), and fixed for histological analysis.

### Progression-free survival and survival curves

Progression-free survival (PFS) of mice was calculated in the subcutaneous experiment. According to the response evaluation criteria in solid tumors (RECIST; ref. 34), a percentage change in tumor size of about 25% from baseline (time of treatments initiation) determined the progression of the disease. A second order polynomial (quadratic) equation was applied to calculate the elapsed time between baseline tumor size and a 25% increase in tumor volume using the GraphPad software (RRID:SCR_002798). Significance was calculated using the Kaplan–Meier survival curve and log-rank (Mantel–Cox) test group and considered when *P* < 0.05.

### MicroCT scan and analysis

To evaluate the growth rate of the orthotopic PMP-PDX, a microCT scan was performed at different time points. Imaging studies were acquired with a Quantum FX microCT Imaging system (Perkin Elmer. 940 Winter St. Waltham, Massachusetts. EEUU), specifically designed for laboratory animal imaging. The acquisition parameters were as follows: Field of View 30 mm (corresponding in a spatial resolution of 0.059 × 0.059 × 0.059 mm), exposure time was 26 seconds, and X-ray voltage 50 kV and amperage 200 uA. The studies were reconstructed based on Feldkamp’s method with dedicated software, resulting in a final matrix composed of 512 × 512 × 512 voxels.

Animals were anesthetized with isoflurane in an oxygen mixture (5% during induction phase, 2% during maintenance) with an air flow set to 0.8 L per minute. The mice received an intraperitoneal injection of an iodine-based radiological contrast agent (Iopamidol 370 75.53 g/100 mL, corresponding to 370 mg of iodine per ml) before the microCT acquisition. This contrast helped to visually distinguish between mucinous tumoral tissue and abdominal soft tissue structures. The animals were moved back to their cages for recovery after image acquisition. All the procedures were performed following institutional ethic committee’s instructions.

Imaging data were analyzed using Slicer software (Slicer.org; ref. 35). Image processing includes a median filtering of the images, with a neighborhood size of 3 voxels. This filtering reduced the noise of the images. Later, a threshold-based segmentation of the mucinous tissue was performed, using a soft tissue density range between −400 and 200 Hounsfield units. Tumor volume was calculated integrating all the segmented areas in each slice into a three-dimensional object.

### Statistics

Statistical analyses were performed with GraphPad Prism 8.1 (RRID:SCR_002798). Data shown represent the mean ± SEM or SD (as indicated in figure legends) of triplicates. Significant differences were assessed using one-way ANOVA with Dunnett’s or Tukey’s multiple comparisons tests or the Kaplan–Meier survival curve and log-rank (Mantel–Cox) test group and considered when *P* < 0.05.

### Data availability

WES data generated in this study are publicly available in Sequence Read Archive (SRA, RRID:SCR_001370) at PRJNA1041789 (https://www.ncbi.nlm.nih.gov/sra/?term=PRJNA1041789).

Digital PCR assays and other data and reagents are available on request through direct communication with the corresponding authors (J. Martínez-Quintanilla and H.G. Palmer).

Raw data behind the figures and tables are available on request through direct communication with the corresponding authors (J. Martínez-Quintanilla and H.G. Palmer).

## Results

### PMP-PDO, PDX, and PDXO are reliable preclinical models to study PMP

**Figure 1.**
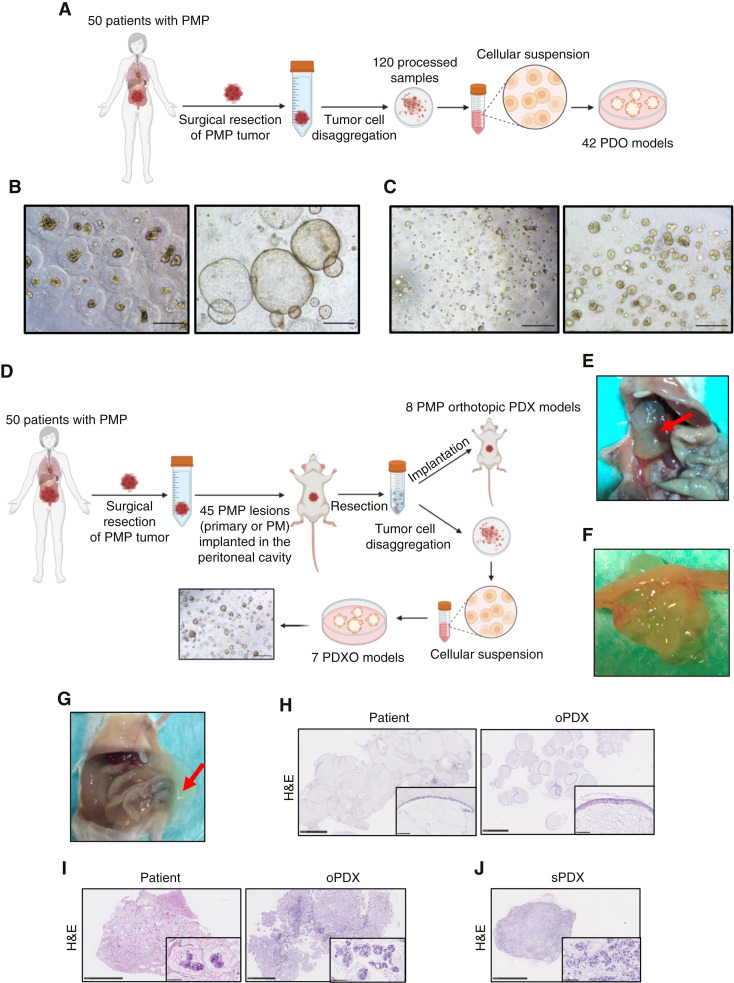
Generation of a collection of PMP-PDO, PDX, and PDXO models. **A,** Design showing the generation of PDO from tumor tissues of patients with PMP. **B,** Pictures of PMP-PDO models after 7 days in culture (PMP18.3, left image and PMP21.5, right image). Scale bar, 500 μm. **C,** Expansion of PMP-PDO models after dissociation with TrypLE Express reagent at day 2 (left) and day 7 (right; PMP5.2). Scale bar, 500 μm **D,** Design showing the generation of PDX and PDXO from tumor tissues of patients with PMP. PM, Peritoneal metastases. **E–G,** Macroscopic images from an orthotopic PMP-PDX (oPDX) models in mice derived from low-grade G1 (**E** and **F**; PMP3.1) or high-grade G3 (**G**; PMP5.1) samples from patients with mucinous carcinoma peritonei. Tumor mass is indicated by red arrows. **H** and **I,** H&E staining from low-grade G1 (**H**; PMP3.1) and high-grade G3 (**I**; PMP5.1) samples from patients with mucinous carcinoma peritonei and associated oPDXs. Scale bar, 2.5 mm and 100 μm. **J,** H&E staining from a subcutaneous PMP-PDX (sPDX) model derived from the sample from patient with high-grade G3 mucinous carcinoma peritonei (PMP5.1). Scale bar, 2.5 mm and 100 μm. (**A** and **D,** Created with BioRender.com.). PMP, Pseudomyxoma peritonei.

A total of 212 fresh samples from 50 patients with PMP who underwent CRS and HIPEC were collected for our study from May 2020 to November 2023. From each patient, primary tumor, when available, and multiple peritoneal metastatic mucinous lesions from PMP were collected (Supplementary Table S3). In around 90% of the cases, histopathological examination showed a neoplastic cellularity lower than 10% (Supplementary Table S3). A total of 120 samples were processed to generate a collection of 42 PMP-PDO models (Fig. 1A). We established five LAMN and one HAMN preclinical models from primary tumor samples and 25 models derived from G1, seven from G2, and four from G3 peritoneal metastatic samples (Table 1; Supplementary Table S3). PMP-PDO cultures were expanded for several passages until they were frozen (Fig. 1B and C). PMP preclinical organoid models preserved the histopathological markers, mucinous secretion, and eventually the resistance to chemotherapy from the original patient (Supplementary Fig. S1A–C; Supplementary Table S4). This last result correlated with the nonresponse to oxaliplatin treatment of the corresponding patient 5 that led to an early recurrence and death in only 3 months (Supplementary Table S3, column PFS).

**Table 1.** Clinicopathological parameters and preclinical models generated from patients with PMP enrolled in the study.

Patient	Sex	Age	PCI	Number of samples	Histological classification	Preclinical models
1	F	72	33	5	LAMN/G1	3
2	F	67	29	6	LAMN/AM	0
3	M	62	27	5	HAMN/G1	4
4	F	76	22	5	LAMN/AM	0
5	F	69	32	4	G3	14
6	M	44	32	6	LAMN/G1	1
7	F	67	5	3	G1	0
8	M	51	2	1	LAMN	0
9	F	39	21	4	AM	0
10	M	52	30	2	G1	0
11	F	77	na	2	G2	0
12	F	59	22	3	G2	1
13	M	58	22	4	G1	0
14	F	49	3	2	G1	2
15	M	56	35	5	G1	2
16	M	76	na	1	LAMN	1
17	M	43	29	6	LAMN/G1	2
18	F	52	18	4	G1	2
19	F	42	31	6	G1	0
20	M	71	3	3	G1/AM	1
21	M	42	33	5	G1	2
22	F	68	16	5	HAMN/AM/G1	0
23	M	70	4	3	AM	0
24	F	60	33	4	LAMN/G1	2
25	M	58	25	4	G1	0
26	M	na	na	6	LAMN/G1	0
27	F	52	0	5	AM	np
28	M	55	3	2	G1	1
29	F	64	34	5	G1	3
30	F	66	26	3	G1	0
31	F	73	4	4	HAMN/AM	0
32	F	66	20	2	G2	1
33	F	72	27	6	HAMN/G2	3
34	M	47	14	6	LAMN/G1	0
35	M	37	34	5	HAMN/G2	2
36	F	46	na	4	LAMN/AM	0
37	M	42	22	5	G1	0
38	F	73	14	4	LAMN/G1	np
39	M	72	10	4	G1	1
40	F	49	19	5	G1	1
41	F	60	27	4	LAMN/G1	2
42	F	73	19	6	G1	1
43	F	65	5	2	G1	1
44	F	63	23	4	LAMN/G1	np
45	M	57	27	4	G1	np
46	M	75	14	4	LAMN/G1	1
47	F	71	31	5	G2	1
48	M	50	39	8	G2	0
49	F	60	19	5	G1	0
53	F	43	na	6	G2	4

Description of the clinicopathological parameters of the patients with PMP included in the study: patient number, sex, age in years, and the Sugarbaker PCI. Moreover, we also listed the number of collected samples, histological classification, and preclinical models generated for each patient.

Abbreviations: AM, acellular mucin; F, female; M, male; na, not applicable.

A total of 45 surgical PMP lesions were orthotopically implanted in the peritoneal cavity of NOD-SCID mice to generate PMP-PDXs and derive their associated organoid models (PMP-PDXO; Fig. 1D; Supplementary Table S3). We established a total of eight PMP orthotopic PDX models, one of them derived from HAMN primary tumor sample, whereas the others were established from G1 (two models), G2 (one model), and G3 (four models) peritoneal metastatic samples. In addition, we successfully generated seven PMP-PDXO models (Table 1; Supplementary Table S3). Orthotopic PMP-PDX models derived from G1 (Fig. 1E and F) or G3 tumors (Fig. 1G) presented large amounts of intra-abdominal mucinous tumor tissue, the main macroscopic feature of patients with PMP. H&E staining (Fig. 1H and I) and IHC markers (Supplementary Fig. S2A) indicated that orthotopic PMP-PDXs faithfully resembled the histological features of the corresponding samples from patients with PMP. Although all PMP-PDX presented high secretion of intra-abdominal mucin, it was differentially distributed between models (Supplementary Fig. S2B). In addition, PMP-PDX models preserved tumor invasiveness in peritoneal organs such as the liver, a phenomenon commonly observed in patients with PMP (Supplementary Fig. S2C). We also established subcutaneous PMP-PDXs by implanting PMP-PDO into the flanks of NOD-SCID mice (Fig. 1J). The implantation rate of these subcutaneous PDX was much lower than the orthotopic models (Supplementary Table S3) and the latency time much longer (data not shown), as previously reported (25).

### Genomic characterization of preclinical models and intra-abdominal mucin biopsy reveal druggable targets in PMP

**Figure 2.**
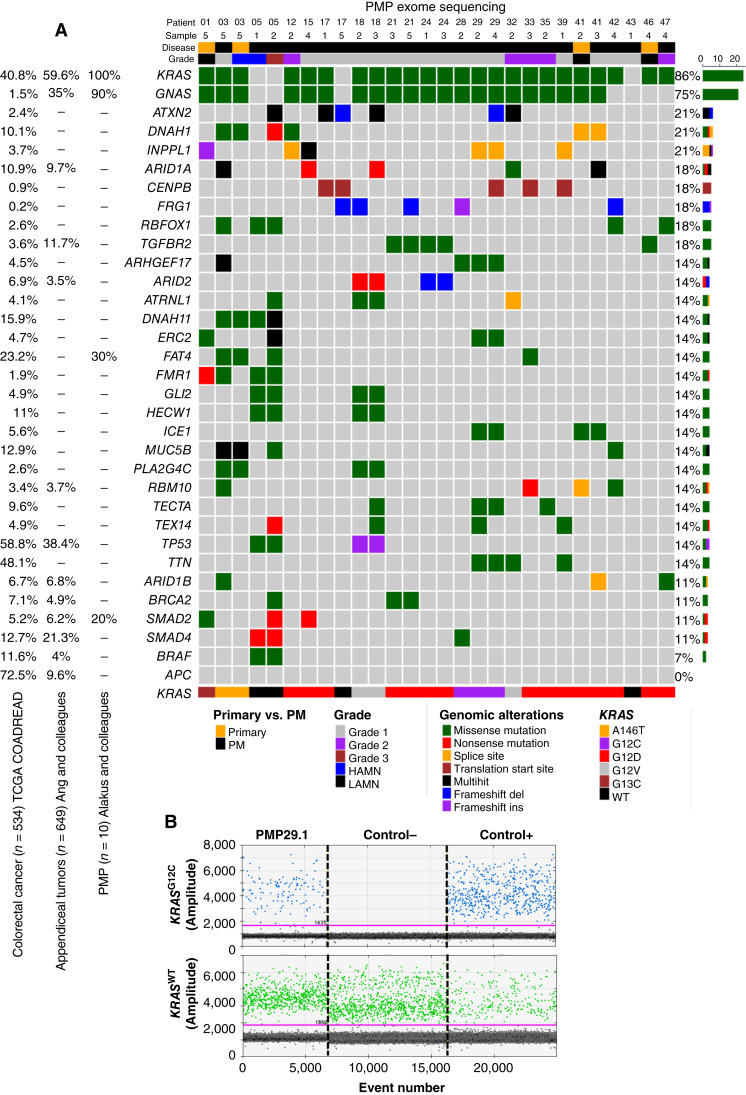
Genomic characterization of preclinical models and intra-abdominal mucin reveal druggable targets in PMP. **A,** Oncoplot from exome sequencing analysis from 28 PMP-PDO or PMP-PDXO models derived from 20 patients. The most common mutated genes in all the models are presented and compared with the cohort of patients with colorectal cancer from the TCGA COADREAD, a cohort of patients with appendiceal tumors from the study of Ang and colleagues and a collection of PMP paraffin samples from the study of Alakus and colleagues. Patient and sample number, primary vs. peritoneal disease, and grade are indicated in the top. Genomic alterations are shown in different colors in the oncoplot. The percentage of PMP preclinical models with the specific mutations is indicated on the right site of the oncoplot. Finally, specific mutations in the *KRAS* gene are shown in the bottom site of the panel. **B,** ddPCR for *KRAS*^G12C^ using genomic DNA extracted from intra-abdominal mucin biopsy of PMP29 (sample PMP29.1; left), negative control (middle), and positive control (right). The study was performed in duplicates. Blue spots and green spots represent positive events for *KRAS*^G12C^ (top graph) and *KRAS*^WT^ genotype (bottom graph), respectively. The threshold line was determined by the control samples to separate the two clusters of negative and positive droplets. PMP, Pseudomyxoma peritonei; CRC, Colorectal cancer; PDO, Patient-derived organoid; PDXO, Patient-derived xenografts organoid; LAMN, low-grade appendiceal mucinous neoplasm; HAMN, high-grade appendiceal mucinous neoplasm; Grade 1, low-grade mucinous carcinoma peritonei; Grade 2, high-grade mucinous carcinoma peritonei; Grade 3, high-grade mucinous carcinoma peritonei with or without signet ring cells.

A total of 28 PMP-PDO or PDXO models derived from 20 different patients with PMP were analyzed by WES to determine their mutational landscape. We show with a summarized oncoplot the most frequently mutated genes comparing our PMP preclinical models with the cohort of patients with colorectal cancer from the TCGA-COADREAD, a cohort of patients with appendiceal tumors from the study of Ang and colleagues (32) and a collection of paraffin samples from patients with PMP from the study of Alakus and colleagues (33). In our study, 86% and 75% of the PMP preclinical models presented *KRAS* and *GNAS* mutations, respectively. From *GNAS*-mutated PMP models we observed two types of mutations: R201H (86%) and R201C (14%). Regarding missense alterations in *KRAS* mutated models we observed five variants: G12D (63%), G12V (13%), G12C (13%), A146 (8%), and G13C (4%; Fig. 2A; Supplementary Table S5) that were validated by PCR using specific oligos (Supplementary Table S2) followed by Sanger sequencing.

Additionally, other cancer-related genes were also mutated in our PMP preclinical models, such as the *BRAF* oncogene (7%); members of the TGF pathway, *TGFBR2* (21%), *SMAD2* (4%), and *SMAD4* (11%); and the tumor suppressor gene *TP53* (14%; Supplementary Table S5). However, *APC*, the tumor suppressor gene most frequently mutated in colorectal cancer, was not altered in any of the PMP models as previously reported (Fig. 2A). Different samples from the same patient were also compared and mostly showed the same mutational landscape (Fig. 2A; Supplementary Table S5). Mutations in *KRAS* and *BRAF* genes were also validated by PCR and Sanger sequencing (data not shown). In summary, our omics analyses indicated several point mutations in specific genes (*KRAS*^G12D^, *KRAS*^G12C^, and *BRAF*^V600E^) in PMP preclinical models that can be targeted with already known small-molecule drugs that bind and inactivate these mutated versions of the proteins (21, 36, 37).

WES analysis of patient sample PMP5.1, that presented 10% of tumor cellularity (Supplementary Table S3), and its corresponding PDXO model resulted in a list of genes that shared the same point mutations (Supplementary Fig. S3A) with the same oncogenic drivers (*BRAF*^V600E^ and *TP53*^G244D^). However, the mean of the VAF from mutated genes was much lower in the patient sample (0.06) than in its corresponding PDXO model (0.5), which is crucial in elucidating the genomic and molecular biology of PMP tumor cells (Supplementary Fig. S3B). The study of oncogenic drivers presents in both samples (*BRAF* and *TP53*) confirmed that VAFs were more than 10 times lower in the patient sample than in the PDXO model. In fact, the subclonal oncogenic mutation in *SMAD4* gene (VAF = 0.19) observed in the PDXO model could not be detected in the corresponding patient sample (Supplementary Fig. S3C), indicating the limitation of WES analysis in PMP patient samples.

Next generation sequencing assays require at least 10% to 20% of tumor cell nuclei to generate reliable somatic mutational data (38). Evaluated by two independent pathologists, more than 90% of our PMP samples did not reach this minimum criterion, excluding them from a proficient WES analysis. To detect and validate the druggable targets (*KRAS*^G12D^, *KRAS*^G12C^, or *BRAF*^V600E^) found in the preclinical models in the corresponding patient’s samples with low cellularity, we explored the use of ddPCR with much higher sensitivity. We studied 66 frozen samples from 15 patients and validated the druggable mutation in 83% of the samples (Supplementary Table S6). We even detected the expected oncogenic mutation in patients’ samples where pathologists indicated a 0% of tumor cellularity. More interestingly, we validated the diagnostic potential of performing ddPCR from intra-abdominal mucin biopsies of six different patients with PMP detecting in all the samples the same druggable mutations as their corresponding preclinical models (Fig. 2B; Supplementary Table S6).

### MAPK signaling pathway inhibition reduces cell viability in PMP preclinical models

The repurposing of FDA-approved drugs can lead to new therapeutic opportunities for rare diseases. We therefore explored the potential benefit of targeted therapies already approved for the treatment of other tumor types in PMP. Previous studies already showed that oncogenic mutations in *KRAS* are much more prevalent than those in *BRAF* gene that are present in only 8% of patients with PMP based on the study by Gleeson and colleagues (10). However, KRAS inhibitors, although showing impacting results in the clinic are still in earlier stage of development compared to BRAF inhibitors that have a pan-solid tumor approval in the US. We believe that the treatment with BRAF inhibitors will be the most rapid option for patients with PMP benefiting from a matched therapy and drugs targeting mutant *KRAS* will come in a later very promising wave. Therefore, as a proof-of-concept, we evaluated the efficacy of encorafenib, a BRAF inhibitor in our *BRAF*^V600E^ PMP preclinical models.

**Figure 3.**
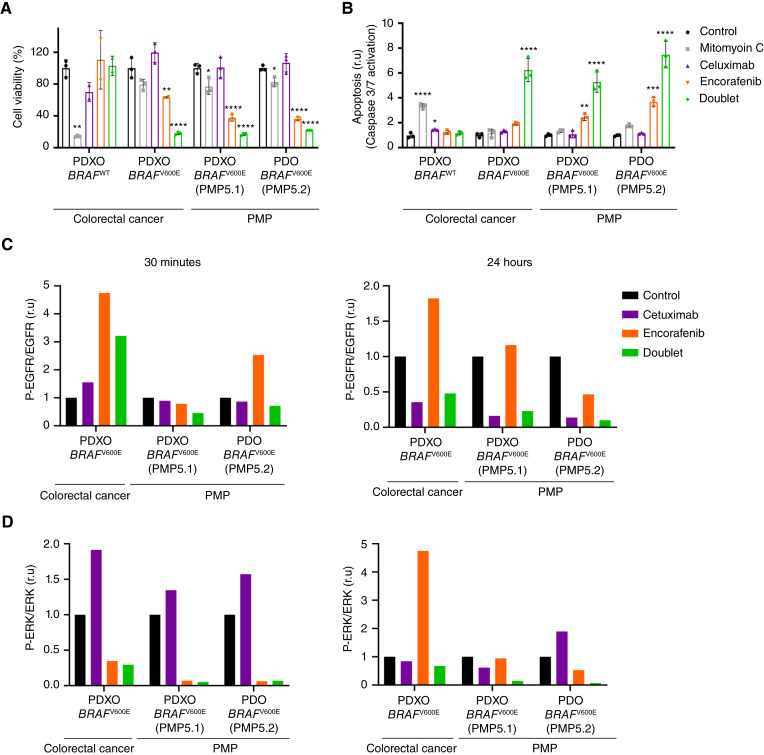
Treatment with BRAF inhibitor is enough to block RAS/ERK signaling pathway and reduce cell viability in PMP models. *BRAF*^WT^ (T70) or *BRAF*^V600E^ (CTAX34) colorectal cancer PDXO and *BRAF*^V600E^ PMP PDXO (PMP5.1) or PDO (PMP5.2) derived from two different peritoneal metastasis from PMP patient 5 treated with vehicle, mitomycin C 150 ng/mL, cetuximab 100 μg/mL, encorafenib 1 μmol/L, doublet (encorafenib + cetuximab). **A** and **B,** Cell viability (**A**) and caspase 3/7 activation (**B**) were measured after 5 days or 48 hours on treatment, respectively. Mean ± SD of triplicates is shown. Significant differences were assessed using one-way ANOVA and Dunnett’s multiple comparisons tests compared to control (*, *P* value < 0.05; **, *P* value < 0.01; ***, *P* value < 0.001; ****, *P* value < 0.0001). **C** and **D,** Western blot analysis of phospho-ERK, phospho-EGFR, ERK, and EGFR was performed after 30 minutes or 24 hours on treatment. Quantification of phospho-EGFR/EGFR (**C**) and phospho-ERK/ERK (**D**) ration in all the samples. r.u, relative units. PMP, Pseudomyxoma peritonei; CRC, Colorectal cancer; PDO, Patient-derived organoid; PDXO, Patient-derived xenografts organoid.

First, we confirmed that encorafenib rapidly blocked the oncogenic MAPK pathway by reducing phospho-ERK (pERK) protein levels in a dose-dependent manner in *BRAF*^V600E^ PMP models (Supplementary Fig. S4A and S4B). Previous work has shown that encorafenib monotherapy is sufficient to downregulate the RAS/BRAF/MAPK signaling pathway and strongly decreases cancer cell viability in *BRAF* mutant melanoma tumors (39, 40). However, very limited response to this same drug was observed in colorectal cancer studies (41, 42) that revealed that *BRAF*^V600E^ tumor cells presented a rapid feedback activation of EGFR expression that mediated resistance to BRAF inhibitors (43, 44). These results justified the use of encorafenib in combination with cetuximab (EGFR inhibitor; doublet) in the preclinical and clinical settings (21). To evaluate whether *BRAF*^V600E^ PMP models behaved as colorectal cancer in response to encorafenib treatment, we treated *BRAF* wild type (*BRAF*^WT^) colorectal cancer (T70), *BRAF*^V600E^ colorectal cancer (CTAX34), and two *BRAF*^V600E^ PMP organoid models derived from two PM lesions from patient 5 (PDXO PMP5.1 and PDO PMP5.2) with control, mitomycin C, cetuximab, encorafenib, or doublet and evaluated cell viability at different time points. Doublet treatment significantly reduced tumor cell viability in *BRAF*^*V600E*^ mutant colorectal cancer as previously described (44), and in both PMP models, but did not have any effect in the *BRAF*^WT^ PDXO cultures (Fig. 3A). Interestingly, encorafenib monotherapy exerted significant therapeutic effect in both *BRAF*^V600E^ PMP models but not in the colorectal cancer model (Fig. 3A) as previously reported (43). On the other hand, mitomycin C showed a slight effect in *BRAF*^V600E^ PMP models (Fig. 3A). Finally, the decrease in cell viability observed in organoid models upon treatment with encorafenib or doublet correlated with an increase in apoptosis mediated by caspase 3/7 activation at 48 hours in *BRAF*^V600E^ colorectal cancer and in both PMP models (Fig. 3B). To elucidate the molecular mechanism of targeted drugs in PMP models, we evaluated EGFR/RAS/MAPK signaling pathway upon treatments. As expected, minor downregulation of phospho-EGFR (pEGFR) shortly (30 minutes) after cetuximab treatment was observed [Supplementary Fig. S5A; Fig. 3C (left)], with a dramatic reduction at 24 hours [Supplementary Fig. S5A; Fig. 3C (right)]. On the other hand, a rapid downregulation of pERK shortly after encorafenib treatment was evidenced [Supplementary Fig. S5A; Fig. 3D (left)]. However, in line with previous publications (44) a signaling upregulation of pERK mediated by an overexpression of EGFR was observed after 24 hours of BRAF blockade and reduced with the doublet in the *BRAF*^V600E^ colorectal cancer PDXO model [Supplementary Fig. S5A; Fig. 3D (right)]. Unlike CRC cultures, *BRAF*^V600E^ PMP models show neither a signaling upregulation of EGFR (expression or activity) nor ERK activation upon encorafenib treatment [Supplementary Fig. S5A; Fig. 3D (right)]. In an independent experiment, encorafenib or binimetinib (MEK inhibitor) alone showed a rapid blockage of the MAPK pathway after 30 minutes in a preclinical model of melanoma, a tumor type with very good response to BRAF inhibitor single agent, as well as in *BRAF*^V600E^ PMP cells (Supplementary Fig. S5B and S5C left). Unexpectedly, encorafenib was even more potent in PMP than in melanoma cells in the long-term inhibition of the oncogenic MAPK pathway as we could not observe reactivation of the ERK phosphorylation, a phenomenon well described as the BRAF paradox (ref. 45; Supplementary Fig. S5B and S5C right). Indeed, this result reinforces the concept of *BRAF*^V600E^ PMP cancer as very addicted to *BRAF* mutation and suggests that encorafenib monotherapy could be a great promise for patients with mutated PMP.

Just to prove that targeted therapy could go beyond BRAF inhibition, we evaluated the efficacy of MRTX1133, a small molecule that specifically inhibits *KRAS*^G12D^, the most prevalent *KRAS* mutation in PMP, alone or in combination with cetuximab (Supplementary Fig. S6). Our results indicated that the KRAS^G12D^ inhibitor reduced tumor cell viability in a dose-dependent manner in a *KRAS*^G12D^ PMP preclinical model derived from a metastatic lesion from patient 53 (PDXO PMP53.1). However, this therapeutic effect was not significantly increased in combination with cetuximab. Colorectal cancer PDXO models either *KRAS*^WT^ (T148) or *KRAS*^G12D^ (CTAX26) as controls showed the expected resistance and response to MRTX1133, respectively.

#### BRAF signaling pathway inhibition suppresses subcutaneous tumor growth in a *BRAF*^V600E^ PMP-PDX model

First, we confirmed that the daily oral administration of encorafenib did not result in any significant body weight reduction or sign of toxicity compared with the vehicle-treated mice (Supplementary Fig. S7A).

**Figure 4.**
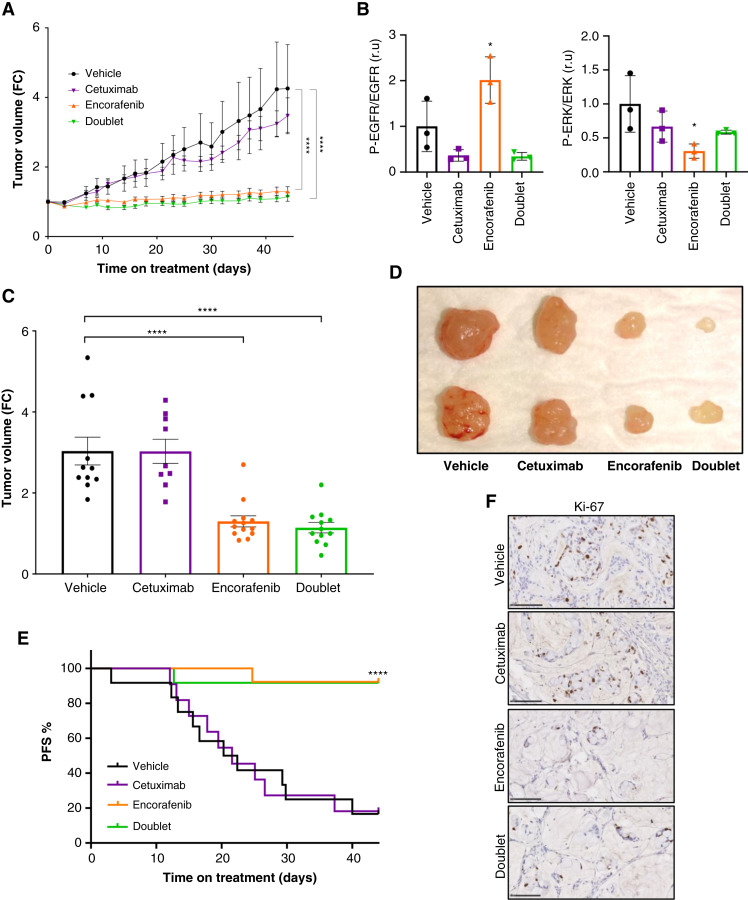
BRAF inhibitor suppresses subcutaneous tumor growth in a *BRAF*^V600E^ PMP-PDX model. *BRAF*^V600E^ mutant PMP-PDX cells from PMP5.1 were subcutaneously implanted in mice and treated with vehicle, intraperitoneal cetuximab (20 mg/kg), oral encorafenib (20 mg/kg), or doublet (*n* = 10–12/group). **A,** Tumor growth curve of the treated mice overtime. Mean ± SEM is shown. **B,** Graphs show the Western blot quantification of pEGFR/EGFR (left) and pERK/ERK (right) ratio from samples of representative excised tumors at day 4 after treatment. Mean ± SD is shown. **C,** Representation of the fold change (FC) in tumor volume for each tumor at day 44 after treatment. Mean ± SEM is shown. Significant differences were assessed using one-way ANOVA and Dunnett’s multiple comparisons tests compared to vehicle group (*, *P* value < 0.05; ****, *P* value < 0.0001; **A** and **C**). **D,** Image of representative tumors from each group at the end of the experiment. **E,** Survival curves represent PFS percentage of each tumor. Mice survival was evaluated by the Kaplan–Meier survival curve and log-rank test group (****, *P* value < 0.0001). **F,** Ki-67 IHC was performed from all tumor samples at the end of the experiment. Images from a representative tumor from vehicle, cetuximab, encorafenib and doublet groups. Scale bar, 100 μm. PMP, Pseudomyxoma peritonei; PDX, Patient-derived xenografts.

Mice bearing subcutaneous *BRAF*^V600E^ PMP tumors were treated with vehicle, intraperitoneal cetuximab, oral encorafenib, or doublet. Both encorafenib and doublet therapy resulted in an equivalent and statistically significant suppression of tumor growth compared with vehicle and cetuximab alone (Fig. 4A). At day 4 after treatment initiation, representative tumors were removed from each group of mice to evaluate the efficacy of treatments blocking the EGFR/RAS/MAPK signaling pathway. Western blot analysis showed that cetuximab, encorafenib, and doublet reduced pEGFR and/or pERK levels (Fig. 4B; Supplementary Fig. S7B). At the end of the experiment, the decrease in tumor growth was homogenous in all tumors of the drug-treated mice (Fig. 4C and D). In addition, PFS analysis indicated that more than 90% of the tumors from the encorafenib or doublet groups presented stabilized disease lasting 44 days upon systemic treatment (Fig. 4E). IHC of tumor sections at the end of the experiment showed reduced tumor cellularity (Supplementary Fig. S8A) and cell proliferation (Fig. 4F) in the encorafenib and doublet compared with the control groups. However, we did not observe differences in mucinous differentiation, mucin secretion (Supplementary Fig. S8B and S8C), or apoptosis (Supplementary Fig. S8D) in the treated compared with the control groups.

#### BRAF signaling pathway inhibition reduces tumor growth in an orthotopic *BRAF*^V600E^ PMP-PDX model

**Figure 5.**
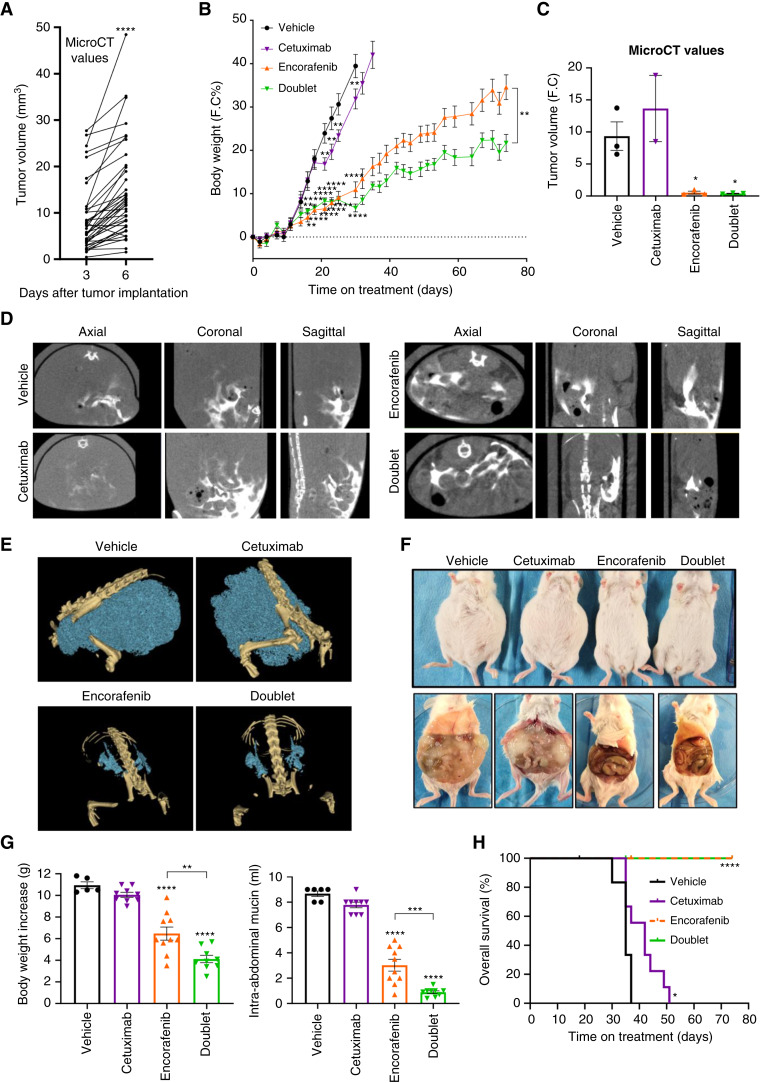
BRAF inhibitor reduces tumor growth in an orthotopic *BRAF*^V600E^ PMP-PDX model. *BRAF*^V600E^ mutant PMP-PDX mucinous tumor tissue from PMP5.1 was implanted intraperitoneally in mice. Animals were scanned using microCT at day 3 and day 6 after tumor implantation. **A,** Representation of paired tumor volume of each mouse at day 3 and 6 after tumor implantation. Significant differences were assessed using Unpaired *t* test (****, *P* < 0.0001). **B–H,** Based on the microCT images, at day 6 animals were divided in four groups and treated with vehicle, intraperitoneal cetuximab (20 mg/kg), oral encorafenib (20 mg/kg), or doublet (*n* = 10–11/group). **B,** Representation of the FC in percentage of body weight the treated mice overtime. Mean ± SEM is shown. Significant differences were assessed using one-way ANOVA and Dunnett’s multiple comparisons tests compared to the vehicle group. Unpaired *t* test to assess significant differences in encorafenib and doublet group was used at the end of experiment. (**, *P* value < 0.01; ***, *P* value < 0.001; ****, *P* value < 0.0001). **C–F,** At day 37, animals were monitored with microCT and a representative animal of each group was euthanized. **C,** FC representation of the tumor volume from microCT images from representative mice from each group (*n* = 2–4). Mean ± SEM is shown. Significant differences were assessed using one-way ANOVA and Dunnett’s multiple comparisons tests compared to vehicle group (*, *P* value < 0.05). **D** and **E,** Axial, coronal, and sagittal microCT images (**D**) and tridimensional rendering of microCT studies (**E**) from a representative mouse from each group. **E,** Pallid blue mucinous tissue volume and pallid yellow bone structures/tissue are shown. **F,** Postmortem images of mice (top) and tumor lesions in the peritoneal cavity (bottom) are shown. **G** and **H,** Animals were euthanized when they reached the end point criteria of 50% of increase in body weight. **G,** Quantification of the weight increase (left) and the volume of mucinous tumor (right) isolated from the intra-abdominal cavity when mice reached the end point criteria. Mean ± SEM is shown. Significant differences were assessed using one-way ANOVA and Tukey’s multiple comparisons tests (**, *P* value < 0.01; ***, *P* value < 0.001; ****, *P* value < 0.0001). **H,** Survival curves from each group. Overall mice survival was evaluated by the Kaplan–Meier survival curve and log-rank test group (****, *P* value < 0.0001). PMP, Pseudomyxoma peritonei; PDX, Patient-derived xenografts.

We next evaluated the efficacy of systemic BRAF-targeted therapy in an orthotopic PMP-PDX model. MicroCT scan images obtained at different time points after tumor implantation confirmed the growth of the mucinous tumors in the peritoneal cavity of implanted mice (Fig. 5A). At this point, microCT measures were used to randomize the mice into four treatment groups: vehicle, intraperitoneal cetuximab, oral encorafenib, and doublet (Supplementary Fig. S9A). We used body weight to monitor the increase of PMP tumor mass over time. A rapid increase in body weight in the vehicle and cetuximab groups was observed whereas a slow increase was seen in the encorafenib and doublet groups (Fig. 5B; Supplementary Fig. S9B). A 50% increase in body weight was considered as an endpoint criterion for euthanizing the animals. All mice from the vehicle group reached this point at day 37. At this time point, microCT images were taken from representative mice and fold change of tumor signal was plotted, showing a massive increase of tumor growth in the control and cetuximab groups compared with the encorafenib and doublet groups (Fig. 5C). MicroCT images (Fig. 5D) and tridimensional rendering (Fig. 5E) of microCT studies from a representative mouse from each group showed that mucinous tumor mass was invading all the peritoneum in the control and cetuximab groups whereas a minimal residual disease was observed in the encorafenib and doublet groups. Abdominal distension and abundant mucinous ascites filling the entire peritoneal cavity was observed in vehicle and cetuximab treated mice (Fig. 5F), correlating with the increase in body weight and the microCT scan measurements. However, at this time, mice from encorafenib and doublet groups presented minimal mucinous ascites in the peritoneal cavity. Evaluation of the mucinous volume extracted from the peritoneal cavity upon necropsy suggested that the increase in body weight in control and cetuximab-treated mice was mainly due to the growth of the PMP tumor (Fig. 5G).

Mice from the vehicle or cetuximab groups reached endpoint criterion at an average of 35 and 42 days, respectively. On the other hand, none of the animals from the encorafenib or doublet groups ever reached the endpoint criterion after 74 days of systemic treatments (Fig. 5H). The survival curve showed that systemic encorafenib targeted therapy provided a profound survival benefit in mice (Fig. 5H). IHC of tumor sections at the end of the experiment showed a decrease in tumor cellularity (Supplementary Fig. S10A) and mucinous secretion (Supplementary Fig. S10B and S10C) in encorafenib and doublet compared to control or cetuximab-treated mice. However, we did not observe a major difference in proliferation (Supplementary Fig. S10D) of the remaining tumor cells at the end of the experiment.

## Discussion

Over the last decade, PDO and PDX models have emerged as optimal preclinical models to study cancer and evaluate new therapeutic treatments (14, 15). While many groups have generated collections of PDO models from different tumor types, this has not yet been achieved for PMP. In our study, we collected 212 fresh samples from 50 patients with PMP and observed that 83% of the tumor samples presented a very low proportion of cancer cells versus stroma cells (0%–5%) as previously described, with only 3% of the cases reaching 20% of tumor cellularity (7). We then selected the most suitable PMP samples based on macroscopic features (120 samples) and generated a collection of 42 PMP-PDO models. Our *in vitro* results showed a success rate of 49% (39 from 79) in the generation of PMP-PDO derived from patients’ samples with at least 1% of tumor cellularity in the H&E section evaluated by two different pathologists. In contrast, our success rate dramatically decreased to 7% (3 from 41) when we processed samples without any tumor cells in the H&E section examined by the pathologists. Our results indicate that a rapid measurement of tumor cell percentage in PMP samples by H&E is crucial to select those cases that will efficiently generate preclinical cultures or xenografts. All PDO, PDX, and PDXO models preserved the histological markers from the original patients and showed to be essential for studying PMP biology as well as evaluating new therapies.

Patient #5 was diagnosed with a massive and very aggressive PMP tumor, histologically classified as a high-grade (G3) mucinous carcinoma peritonei with a poor prognosis. The extension of the tumor mass probably destroyed the appendix and complicated the identification of the appendiceal primary tumor. The primary tumor is not frequently found in patients with these aggressive types of PMP. Therefore, classification of these patients is based on radiological and histopathological evaluation of the peritoneal metastatic samples (2). Moreover, to discard the presence of malignant cells in the colon that could originate PM, a right hemicolectomy was performed. Patient #5 did not show neoplastic cells in the colon and was diagnosed as PMP with unknown origin. *In vitro* BRAF experiments were performed in two preclinical models derived from PM from patient 5. Unfortunately, a formal comparison between primary and metastatic tumors in response to BRAF inhibitors could not be performed since we did not have access to the primary tumor. However, evaluating the therapeutic effect in metastatic lesions is a reasonable option as systemic targeted therapies may potentially be used in the future only in patients with PMP that progress to CRS/HIPEC and therefore in most cases the disease would be exclusively metastatic, as primary tumor will not be present in most cases.

Our genomic data revealed that the oncogenic *BRAF*^V600E^ mutation was present in all four high-grade (G3) mucinous carcinoma peritonei collected from patient 5. *BRAF* mutations, although a rare event present in only 8% of patients with PMP (10), have shown to be enriched in high-grade PMP with SRC compared with samples from patients with low-grade PMP (9, 10). Over recent years, different BRAF inhibitors have been approved by the FDA for the treatment of metastatic melanoma and have shown improved median PFS as a single agent (46). However, preclinical models of *BRAF*^V600E^ colorectal cancer shown that BRAF inhibition causes rapid feedback activation of RAS/MAPK oncogenic signaling pathway through inducing the overexpression of EGFR (43, 44) and required the combination with anti-EGFR monoclonal antibodies to observe antitumoral activity in the clinic (21). Interestingly, our results indicated that PMP models did not present this feedback loop activation through EGFR observed in colorectal cancer. In addition, PMP cells also did not show the BRAF paradox upon treatment with BRAF inhibitors, when compared with *BRAF*^V600E^ melanoma cells (45). Altogether, *in vitro* results and *in vivo* subcutaneous experiments confirmed that *BRAF*^V600E^ PMP tumors are very addicted to *BRAF* mutation and encorafenib alone was sufficient to exert a potent reduction of cancer cell viability and tumor growth. However, orthotopic experiments showed that intraperitoneal cetuximab could improve the therapeutic effect of systemic encorafenib monotherapy. These results could be explained because cetuximab was administered intraperitoneally, directly where the PMP tumors grew, and therefore more bioactive in an orthotopic setting versus a subcutaneous one. Nevertheless, oral encorafenib showed strong and durable therapeutic benefit in both *in vivo* experiments and suggest for the first time that the systemic administration of targeted therapies could benefit patients with PMP.

Genomic characterization revealed *GNAS* mutations (R201H and R201C) in 71% of our PMP models. *GNAS* mutations have been associated with abnormal mucinous secretion and an alternative mechanism of Wnt/β-catenin signaling activation in different cancer types (47). Previous studies from Flatmark and colleagues explored the potential of a peptide vaccine targeting mutated *GNAS* in patients with PMP with very promising results (48). On the contrary, *APC* mutations, the most frequent alterations observed in colorectal cancer, that cause canonical oncogenic Wnt/β-catenin signaling activation, were not detected in any of the PMP preclinical models analyzed. Moreover, our genomic analysis identified common missense alterations in *KRAS*. In a landmark study, patients with *KRAS*^G12C^ mutant lung cancer showed clinical benefit from sotorasib, a first-in-class small-molecule inhibitor that can specifically bind and block the mutant *KRAS*^G12C^ (37, 49). Most recently, preclinical studies in pancreatic cancer have evaluated the therapeutic efficacy of the potent and selective KRAS^G12D^ inhibitor MRTX1133 (36, 50). We observed that 75% of PMP tumors from our collection were driven by *KRAS*^G12D^ or *KRAS*^G12C^ mutations. In addition, our results showed therapeutic effect of MRTX1133 in a *KRAS*^G12D^ PMP preclinical model *in vitro* opening new avenues for evaluating KRAS-targeted therapies in patients with PMP. We consequently envision that our collection of PMP preclinical models will be essential in assessing the efficacy of these therapeutic drugs and ultimately translating our findings into new treatment opportunities for these patients.

The genomic analysis of liquid biopsies has revolutionized clinical practice in many hospitals worldwide, providing oncologists with a noninvasive approach to obtain valuable data for determining the optimal treatment strategy for each patient. Our results suggest that genomic analysis of an also easily accessible intra-abdominal mucin biopsy by ddPCR can be used as a diagnostic tool to detect *KRAS*^G12D^, *KRAS*^G12C^, and *BRAF*^V600E^ druggable mutations in patients with PMP. ddPCR is a rapid and economically sustainable technology that could be implemented as a screening test in the clinical management of patients with PMP in the near future.

**Figure 6.**
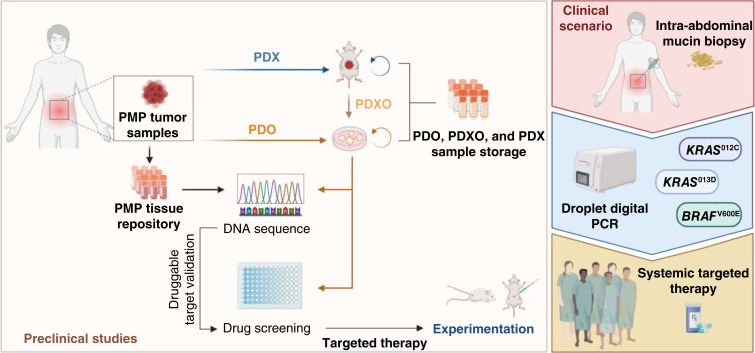
Schematic representation of the results of the study and the potential clinical translation. Design showing the generation of a unique collection of preclinical models derived from PMP tumor samples used in this study. Genomic characterization of these models revealed druggable targets that were validated *in vitro* and *in vivo*. In a clinical scenario, ddPCR from intra-abdominal mucin biopsies of patients with PMP could be used as a diagnostic tool to detect these druggable mutations and propose an individual matched systemic targeted therapy for each patient (Created with BioRender.com.). PMP, Pseudomyxoma peritonei; PDXO, Patient-derived xenografts organoid.

Genomic characterization of our collection of PMP preclinical models revealed druggable mutations. The use of these models demonstrated for the first time that precision oncology could improve outcomes in PMP. In a clinical scenario, this study paves the way to identify by ddPCR those patients who would most likely benefit from promising and potent systemic therapies targeting matched dysregulated drivers of the RAS/MAPK oncogenic pathway (Fig. 6).

### Conclusions

Our study has demonstrated that intra-abdominal mucin biopsy can be used in the clinic for detecting druggable targets in patients with PMP and guide the selection of molecular targeted therapies. As a proof of concept, the systemic pharmacological inhibition of oncogenic BRAF signaling could be an effective therapeutic opportunity for patients with PMP presenting a *BRAF*^V600E^ mutation. Moreover, this aggressive type of PMP is more frequently associated with high-grade mucinous carcinoma peritonei with signet ring cells in patients with recurrent disease. These patients have a dismal prognosis, without current effective treatments. Our results pave the way for extending the promise of precision oncology to patients with PMP who could for the first time derive benefit from personalized matched targeted therapies.

## Supplementary Material

Supplementary Figure 1 Supplementary Figure 1. PMP preclinical organoid models preserve histological markers and chemotherapy resistance from the original patient sample and present different patterns of mucinous secretion.

Supplementary Figure 2 Supplementary Figure 2. PMP-PDX models resemble histological features of the original patient sample with massive mucinous secretion and preserve invasiveness.

Supplementary Figure 3 Supplementary Figure 3. PMP5.1-PDX model preserves oncogenic mutations from the original patient sample.

Supplementary Figure 4 Supplementary Figure 4. BRAF inhibitor reduces Phospho-ERK in BRAFV600E mutant PMPPDO and PDXO models in a dose dependent manner.

Supplementary Figure 5 Supplementary Figure 5. Treatment with BRAF inhibitor is enough to prolong RAS/ERK signaling pathway blockage in PMP models.

Supplementary Figure 6 Supplementary Figure 6. Treatment with KRASG12D inhibitor reduces cell viability in KRASG12D mutant PMP-PDXO model in a dose dependent manner.

Supplementary Figure 7 Supplementary Figure 7. BRAF signaling pathway inhibition in a subcutaneous BRAFV600E PMP-PDX model.

Supplementary Figure 8 Supplementary Figure 8. Treatment with BRAF inhibitors reduces tumor cell proliferation in subcutaneous BRAFV600E PMP-PDX tumors without impacting in apoptosis.

Supplementary Figure 9 Supplementary Figure 9. BRAF inhibitors reduce tumor mass in an orthotopic BRAFV600E PMP-PDX model.

Supplementary Figure 10 Supplementary Figure 10. BRAF inhibitor treatment impacts in the mucinous structure of PMP tumors.

Supplementary Table 1 Supplementary Table 1. Primary and secondary antibodies, commercial provider, reference, and dilution used for WB or IHC.

Supplementary Table 2 Supplementary Table 2. Primer sets of target genes.

Supplementary Table 3 Supplementary Table 3. Expanded clinicopathological parameters and preclinical models established from PMP patients enrolled in the study.

Supplementary Table 4 Supplementary Table 4. Correlation of PMP markers in patient samples and their corresponding organoid model.

Supplementary Table 5 Supplementary Table 5. Mutational status of the PMP organoid models used in the study.

Supplementary Table 6 Supplementary Table 6. Validation of druggable targets in the tumor samples from PMP patients by droplet digital PCR (ddPCR).
