# Supplementary material for: Precision Oncology and Systemic Targeted Therapy in Pseudomyxoma Peritonei
Source: Clin Cancer Res. 2024 Jul 11;30(18):4082–99. doi: 10.1158/1078-0432.CCR-23-4072 (PMC11393541; doi:10.1158/1078-0432.CCR-23-4072)
Supplement: Supplementary Figure 1 — PMP preclinical organoid models preserve histological markers and chemotherapy resistance from the original patient sample and present different patterns of mucinous secretion. [file ccr-23-4072_supplementary_figure_1_suppsf1.pdf]

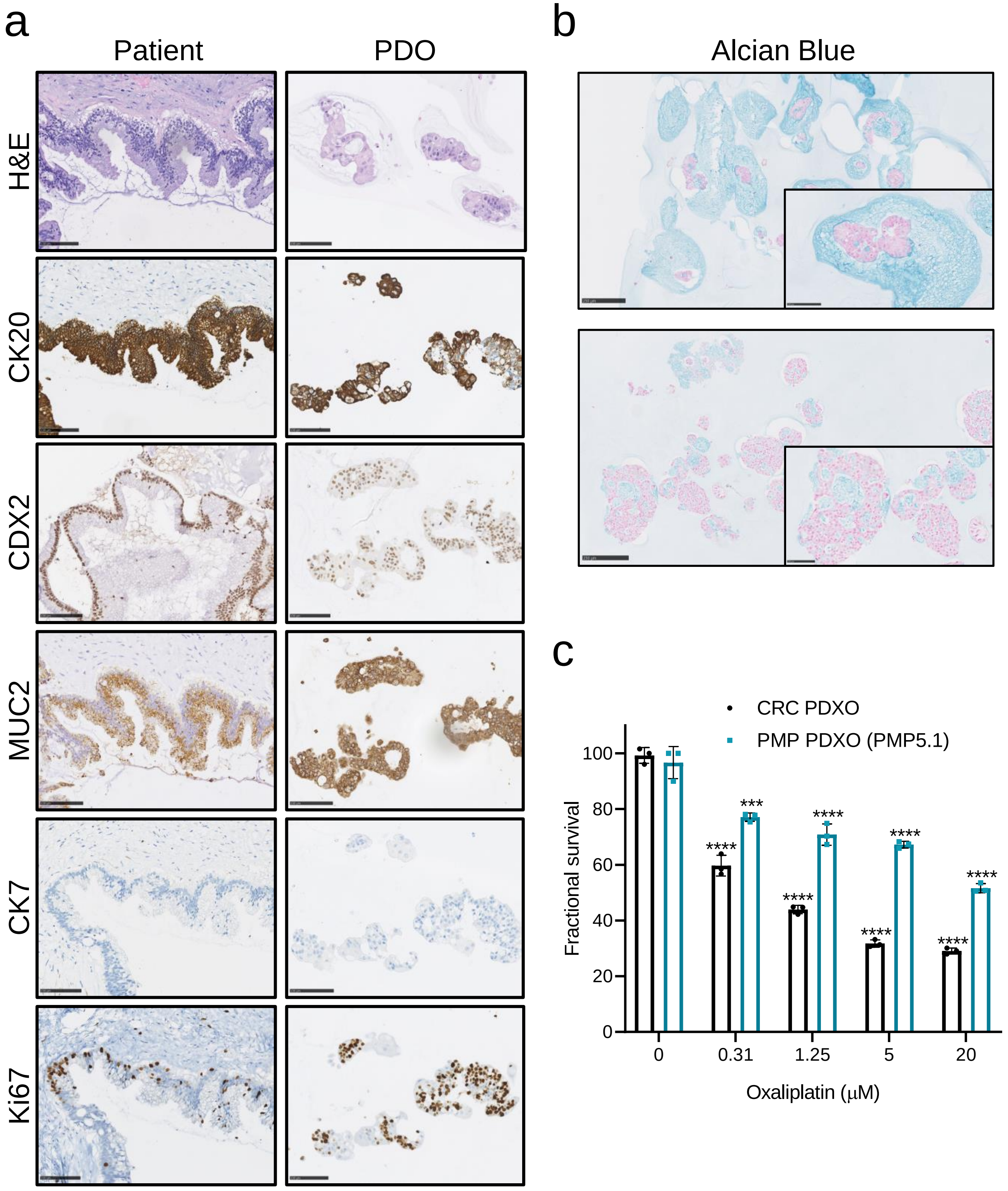

a
Patient
PDO
H&E
CK20
CDX2
MUC2
CK7
Ki67
b
Alcian Blue
c
CRC PDXO
PMP PDXO (PMP5.1)
Fractional survival
0
20
40
60
80
100
0
0.31
1.25
5
20
Oxaliplatin (μM)
***
****

Supplementary Figure 1: PMP preclinical organoid models preserve histological markers and chemotherapy resistance from the original patient sample and present different patterns of mucinous secretion. a) Hematoxylin & Eosin (H&E) and immunohistochemistry of different PMP markers (CK20, CDX2, MUC2, CK7 and Ki67) in paired samples from patient and PDO (PMP18.3). Scale bar 100 µm. **b**) Alcian blue staining of PMP-PDO models derived from G1 (PMP18.3) (**upper image**) or G3 (PMP5.2) (**bottom image**) mucinous carcinoma peritonei samples. Scale bar 250 µm and 100 µm. **c**) CRC PDXO (CTAX26) as control and PMP PDXO (PMP5.1) were treated with oxaliplatin at different doses. Cell viability was measured after 5 days on treatment. Mean ± SD of triplicates is shown. Significant differences were assessed using one-way ANOVA and Dunnett's multiple comparisons tests compared to control (***p value < 0.001, ****p value < 0.0001). PMP = Pseudomyxoma peritonei, CRC = Colorectal cancer, PDO = Patient-derived organoid, PDXO = Patient-derived xenografts organoid.
