# Supplementary material for: Precision Oncology and Systemic Targeted Therapy in Pseudomyxoma Peritonei
Source: Clin Cancer Res. 2024 Jul 11;30(18):4082–99. doi: 10.1158/1078-0432.CCR-23-4072 (PMC11393541; doi:10.1158/1078-0432.CCR-23-4072)
Supplement: Supplementary Figure 2 — PMP-PDX models resemble histological features of the original patient sample with massive mucinous secretion and preserve invasiveness. [file ccr-23-4072_supplementary_figure_2_suppsf2.pdf]

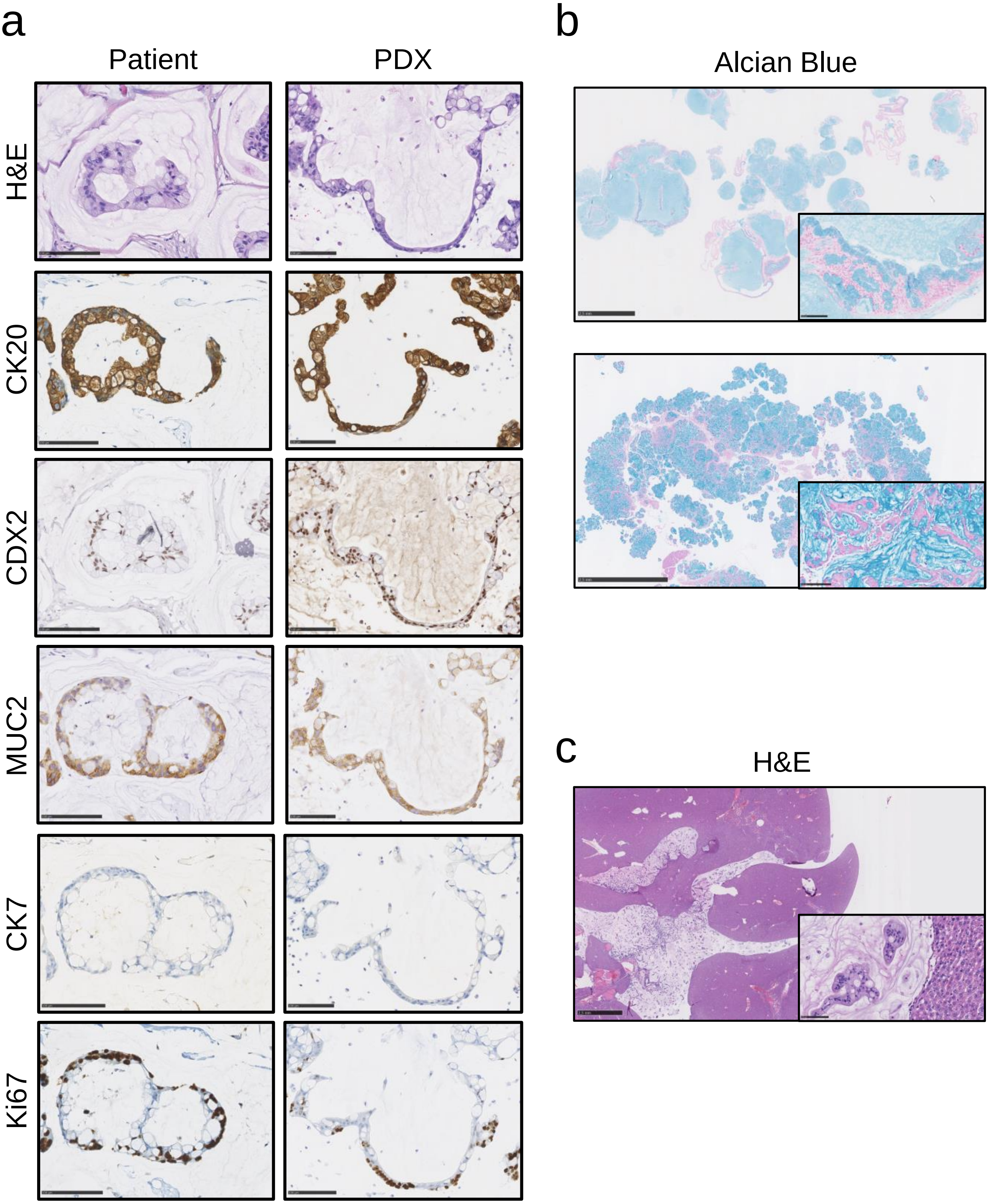

a
Patient
PDX
H&E
CK20
CDX2
MUC2
CK7
Ki67
b
Alcian Blue
c
H&E

Supplementary Figure 2: PMP-PDX models resemble histological features of the original patient sample with massive mucinous secretion and preserve invasiveness. **a**) Hematoxylin & Eosin (H&E) and immunohistochemistry of different PMP markers (CK20, CDX2, MUC2, CK7 and Ki67) in a paired PMP patient sample and PDX model (PMP5.1). Scale bar 100 µm. **b**) Alcian blue staining of PMP-PDXO model derived from G1 (PMP3.5) (**above**) or G3 (**below**) (PMP5.1) PMP peritoneal tumor sample. Scale bar 250 µm and 100 µm. **c**) H&E staining from a mouse liver bearing PMP-PDX G3 peritoneal model (PMP5.3). Scale bar 2.5 mm and 100 µm. PMP = Pseudomyxoma peritonei, PDX = Patient-derived xenografts.
