# Supplementary material for: Precision Oncology and Systemic Targeted Therapy in Pseudomyxoma Peritonei
Source: Clin Cancer Res. 2024 Jul 11;30(18):4082–99. doi: 10.1158/1078-0432.CCR-23-4072 (PMC11393541; doi:10.1158/1078-0432.CCR-23-4072)
Supplement: Supplementary Figure 3 — PMP5.1-PDX model preserves oncogenic mutations from the original patient sample. [file ccr-23-4072_supplementary_figure_3_suppsf3.pdf]

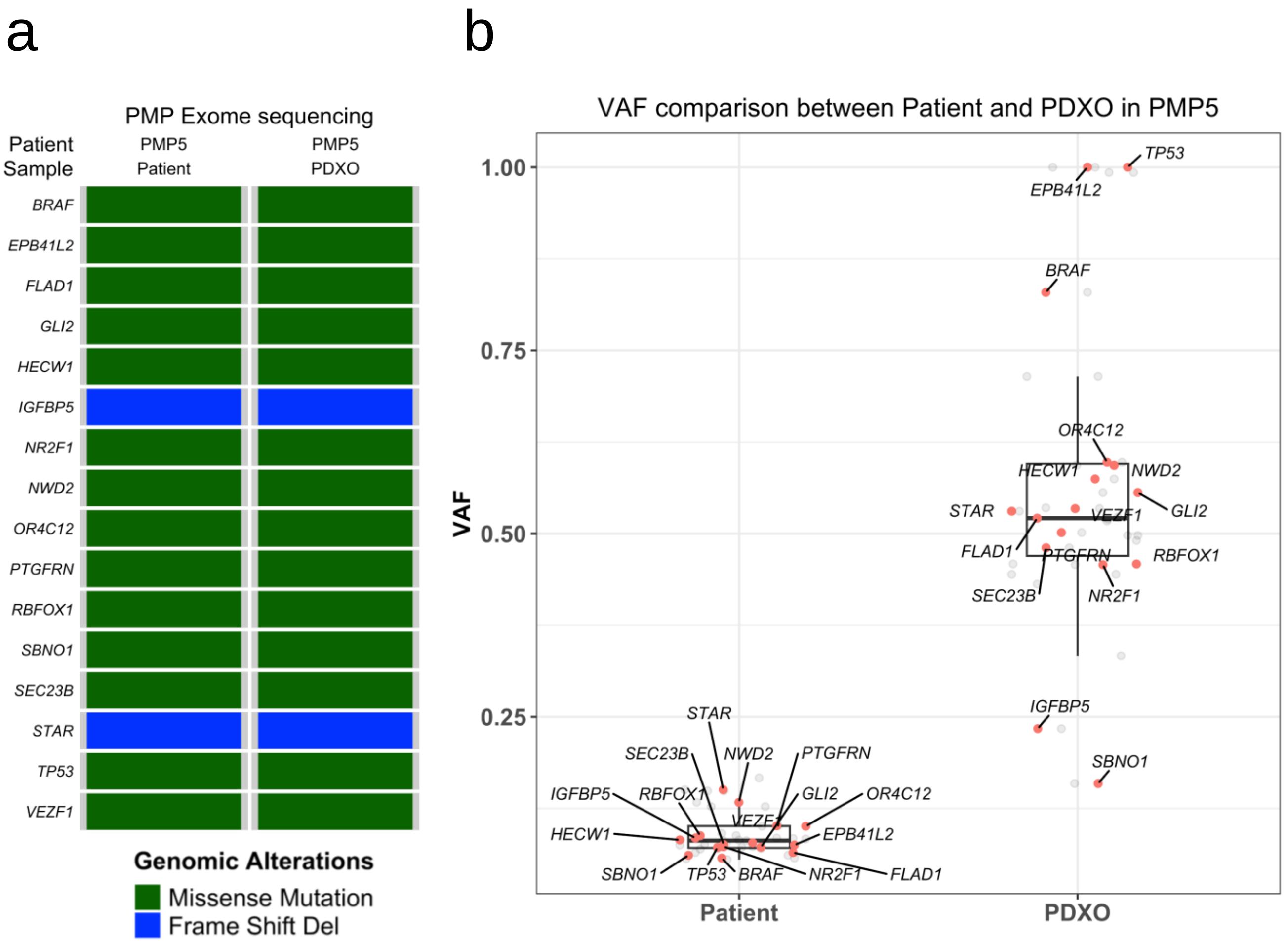

c

Gene name	Genomic alteration	Base Change	AA Change	Oncogenesis relation	Patient VAF	PDXO VAF
BRAF	missense variant	c.1799T>A	p.Val600Glu	Oncogene	0,057	0,83
TP53	missense variant	c.731G>A	p.Gly244Asp	Tumor suppressor gene	0,072	1
SMAD4	stop gained	c.1612G>T	p.Glu538*	Tumor suppressor gene	not detected	0,19

Supplementary Figure 3: PMP5.1-PDX model preserves oncogenic mutations from the original patient sample. a) Oncoplot from exome sequencing analysis from patient sample PMP5.1 and its corresponding PDXO model showing shared point mutated genes. **b**) Plot showing variant allele frequency (VAF) from shared point mutated genes in both samples. **c**) Detection of cancer related genes mutated in the PDXO model and the corresponding patient sample. The name of the gene, genomic alteration, nucleotide base changed, amino acid (AA) change, oncogenesis relation and the VAF, if applicable, from the patient and the PDXO are listed. PMP = Pseudomyxoma peritonei, PDX = Patient-derived xenografts, PDXO = Patient-derived xenografts organoid.
