# Supplementary material for: Precision Oncology and Systemic Targeted Therapy in Pseudomyxoma Peritonei
Source: Clin Cancer Res. 2024 Jul 11;30(18):4082–99. doi: 10.1158/1078-0432.CCR-23-4072 (PMC11393541; doi:10.1158/1078-0432.CCR-23-4072)
Supplement: Supplementary Figure 4 — BRAF inhibitor reduces Phospho-ERK in BRAFV600E mutant PMPPDO and PDXO models in a dose dependent manner. [file ccr-23-4072_supplementary_figure_4_suppsf4.pdf]

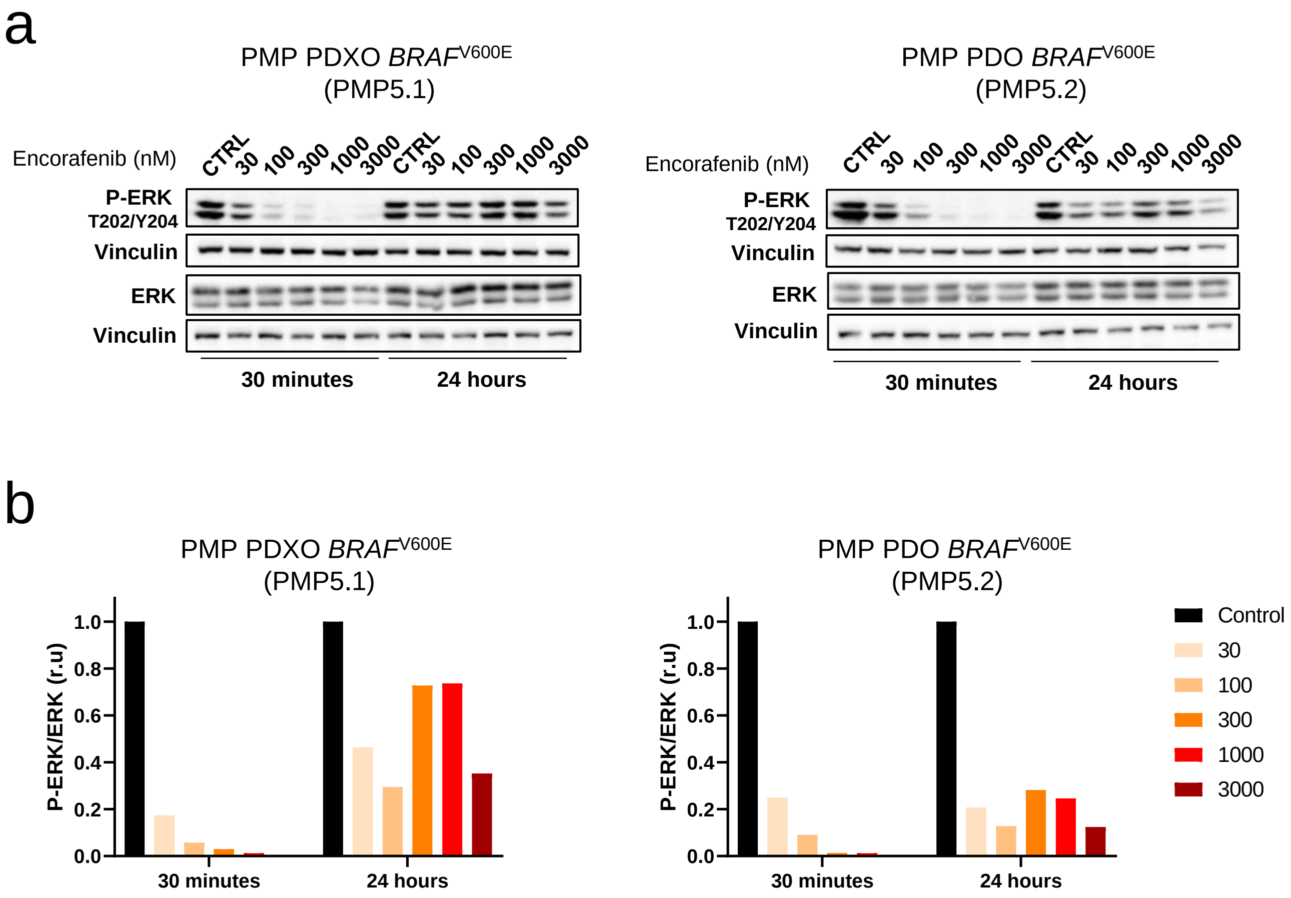

Supplementary Figure 4: BRAF inhibitor reduces Phospho-ERK in *BRAF*^{V600E} mutant PMP-PDO and PDXO models in a dose dependent manner. (a-b) Western blot analysis of phospho-ERK and ERK in *BRAF*^{V600E} PMP PDXO (PMP5.1) (**left**) or PDO (PMP5.2) (**right**) (**a**) treated with vehicle or increasing doses of encorafenib for 30 minutes or 24 hours. **b**) Quantification of phospho-ERK/ERK ration in all the samples. r.u = relative units, PMP = Pseudomyxoma peritonei, PDO = Patient-derived organoid, PDXO = Patient-derived xenografts organoid
