# Supplementary material for: Precision Oncology and Systemic Targeted Therapy in Pseudomyxoma Peritonei
Source: Clin Cancer Res. 2024 Jul 11;30(18):4082–99. doi: 10.1158/1078-0432.CCR-23-4072 (PMC11393541; doi:10.1158/1078-0432.CCR-23-4072)
Supplement: Supplementary Figure 5 — Treatment with BRAF inhibitor is enough to prolong RAS/ERK signaling pathway blockage in PMP models. [file ccr-23-4072_supplementary_figure_5_suppsf5.pdf]

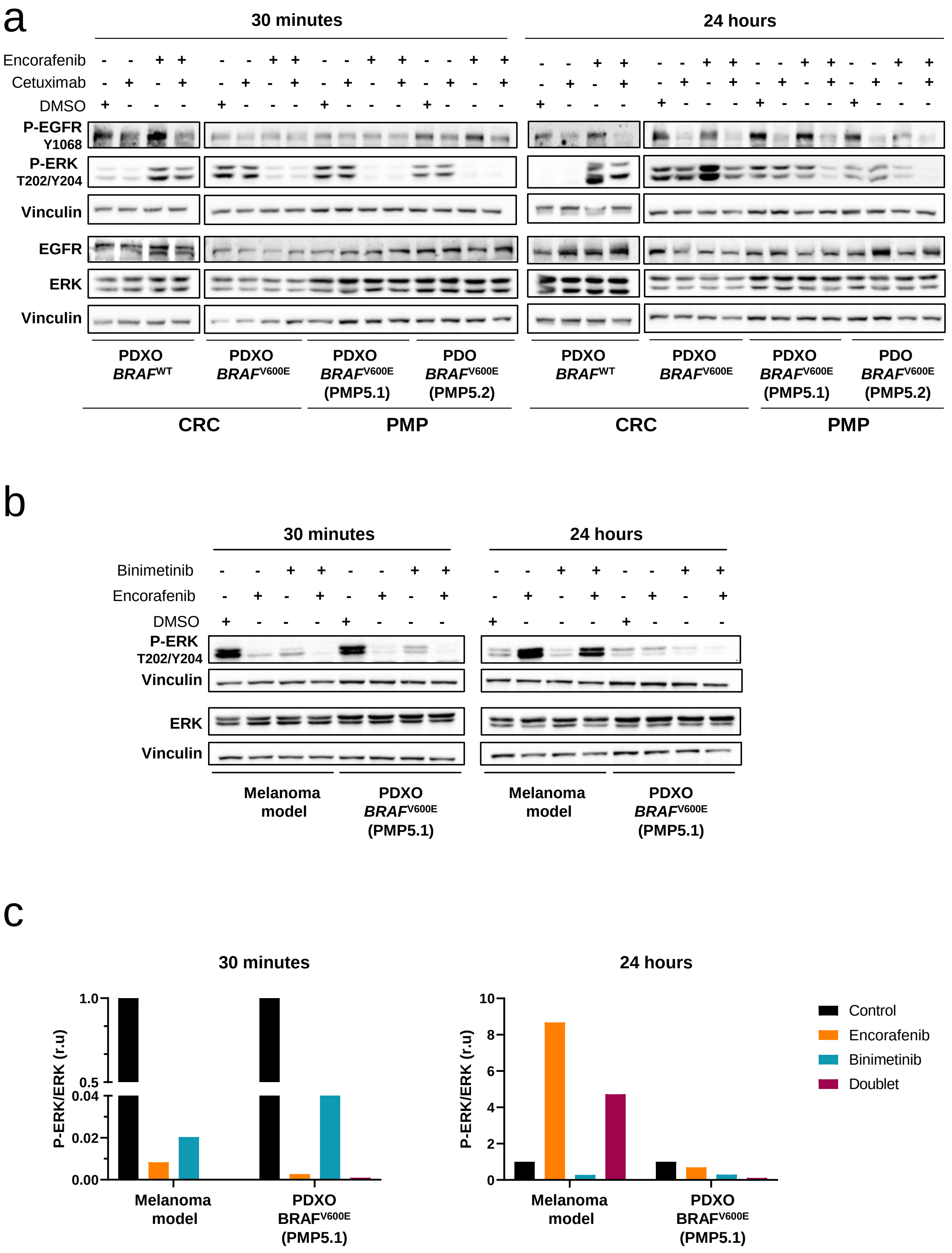

a
30 minutes
24 hours
Encorafenib
Cetuximab
DMSO
P-EGFR Y1068
P-ERK T202/Y204
Vinculin
EGFR
ERK
Vinculin
PDXO BRAF^WT
PDXO BRAF^V600E
PDXO BRAF^V600E (PMP5.1)
PDO BRAF^V600E (PMP5.2)
CRC
PMP
b
30 minutes
24 hours
Binimetinib
Encorafenib
DMSO
P-ERK T202/Y204
Vinculin
ERK
Vinculin
Melanoma model
PDXO BRAF^V600E (PMP5.1)
c
30 minutes
24 hours
P-ERK/ERK (r.u)
1.0
0.5
0.04
0.02
0.00
10
8
6
4
2
0
Melanoma model
PDXO BRAF^V600E (PMP5.1)
Control
Encorafenib
Binimetinib
Doublet

Supplementary Figure 5: Treatment with BRAF inhibitor is enough to prolong RAS/ERK signalling pathway blockage in PMP models. ***a)*** *BRAF*^{WT} (T70) or *BRAF*^{V600E} (CTAX34) CRC PDXO and *BRAF*^{V600E} PMP PDXO (PMP5.1) or PDO (PMP5.2) were treated with vehicle, mitomycin C 150 ng/ml, cetuximab 100 µg/ml, encorafenib 1 µM, doublet (encorafenib + cetuximab). Western blot analysis of phospho-ERK, phospho-EGFR, ERK and EGFR was performed after 30 minutes or 24 hours on treatment. **b-c**) *BRAF*^{V600E} melanoma model (A375 organoids) and *BRAF*^{V600E} PMP PDXO (PMP5.1) were treated with vehicle, encorafenib 1µM, binimetinib 200nM, doublet (encorafenib + binimetinib). Western Blot analysis of phospho-ERK and ERK was performed after 30 minutes or 24 hours on treatment (**b**) and phospho-ERK/ERK ration was quantified in all the samples (**c**). r.u = relative units, PMP = Pseudomyxoma peritonei, CRC = Colorectal cancer, PDO = Patient-derived organoid, PDXO = Patient-derived xenografts organoid.
