# Supplementary material for: Precision Oncology and Systemic Targeted Therapy in Pseudomyxoma Peritonei
Source: Clin Cancer Res. 2024 Jul 11;30(18):4082–99. doi: 10.1158/1078-0432.CCR-23-4072 (PMC11393541; doi:10.1158/1078-0432.CCR-23-4072)
Supplement: Supplementary Figure 6 — Treatment with KRASG12D inhibitor reduces cell viability in KRASG12D mutant PMP-PDXO model in a dose dependent manner. [file ccr-23-4072_supplementary_figure_6_suppsf6.pdf]

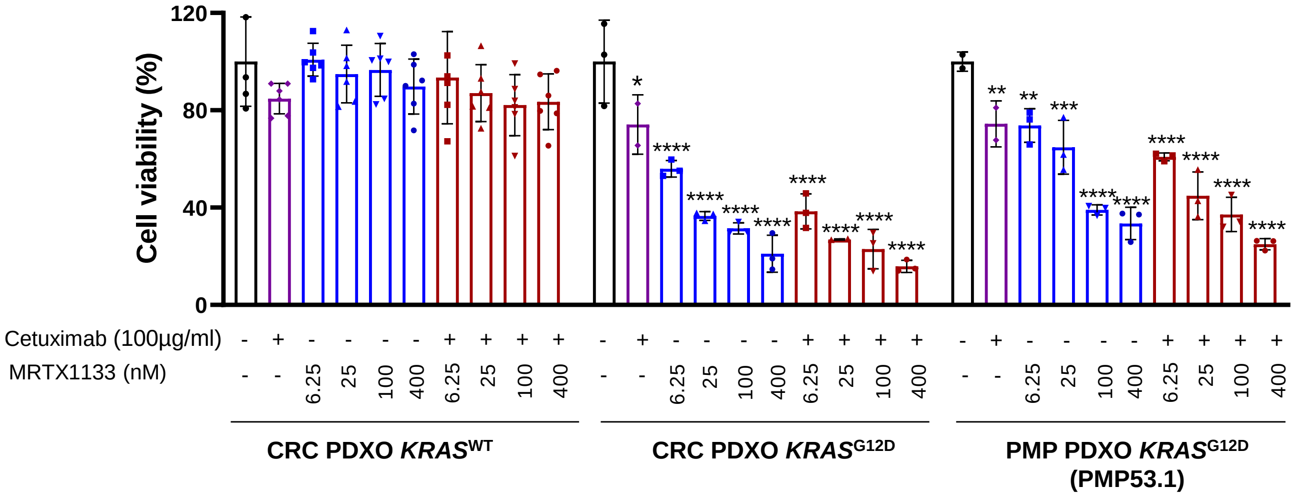

Supplementary Figure 6: Treatment with KRAS^{G12D} inhibitor reduces cell viability in *KRAS*^{G12D} mutant PMP-PDXO model in a dose dependent manner. *KRAS*^{WT} (T148) or *KRAS*^{G12D} (CTAX26) CRC PDXO and *KRAS*^{G12D} PMP PDXO (PMP53.1) were treated with vehicle, cetuximab 100 µg/ml, MRTX1133 at different doses or doublet (MRTX1133 + cetuximab). Cell viability was measured after 5 days on treatment. Mean ± SD of triplicates is shown. Significant differences were assessed using one-way ANOVA and Dunnett's multiple comparisons tests compared to control (*p value < 0.05, **p value < 0.01, ***p value < 0.001, ****p value < 0.0001). PMP = Pseudomyxoma peritonei, CRC = Colorectal cancer, PDXO = Patient-derived xenografts organoid.
