# Supplementary material for: Precision Oncology and Systemic Targeted Therapy in Pseudomyxoma Peritonei
Source: Clin Cancer Res. 2024 Jul 11;30(18):4082–99. doi: 10.1158/1078-0432.CCR-23-4072 (PMC11393541; doi:10.1158/1078-0432.CCR-23-4072)
Supplement: Supplementary Figure 7 — BRAF signaling pathway inhibition in a subcutaneous BRAFV600E PMP-PDX model. [file ccr-23-4072_supplementary_figure_7_suppsf7.pdf]

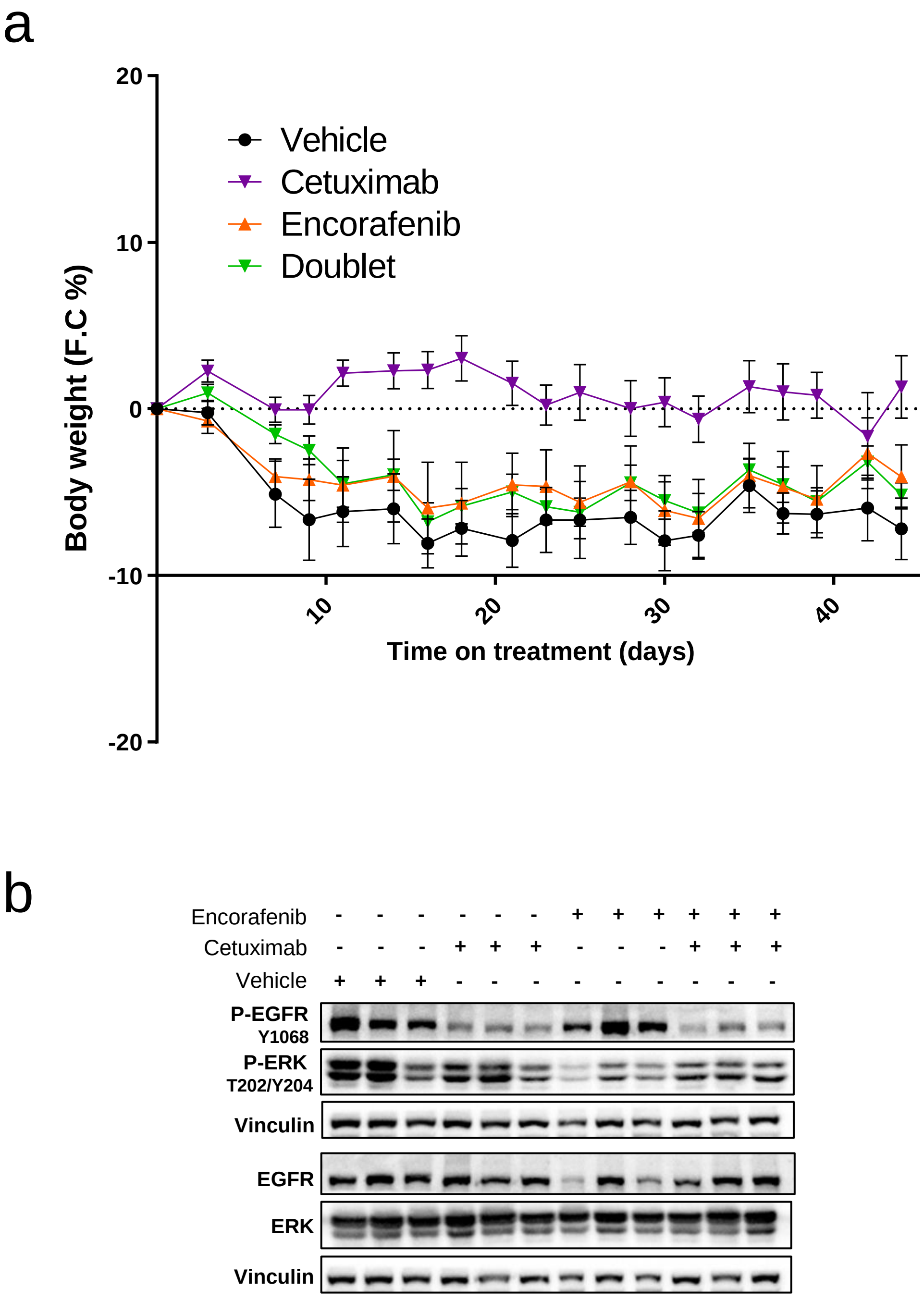

Supplementary Figure 7: BRAF signaling pathway inhibition in a subcutaneous *BRAF*^{V600E} PMP-PDX model. a) Representation of the fold change (F.C) in percentage of body weight of mice treated with vehicle, cetuximab, encorafenib or doublet after 44 days on treatment. **b)** *BRAF*^{V600E} mutant PMP-PDX cells from PMP5.1 were subcutaneously implanted in mice and treated with vehicle, intraperitoneal cetuximab (20 mg/kg), oral encorafenib (20 mg/kg) or doublet (n= 10-12/group). Western blot analysis of phospho-ERK, ERK, phospho-EGFR and EGFR of total cell lysates of representative excised tumours at day 4 after treatment are shown. PMP = Pseudomyxoma peritonei, PDX = Patient-derived xenografts.
