# Supplementary material for: Precision Oncology and Systemic Targeted Therapy in Pseudomyxoma Peritonei
Source: Clin Cancer Res. 2024 Jul 11;30(18):4082–99. doi: 10.1158/1078-0432.CCR-23-4072 (PMC11393541; doi:10.1158/1078-0432.CCR-23-4072)
Supplement: Supplementary Figure 8 — Treatment with BRAF inhibitors reduces tumor cell proliferation in subcutaneous BRAFV600E PMP-PDX tumors without impacting in apoptosis. [file ccr-23-4072_supplementary_figure_8_suppsf8.pdf]

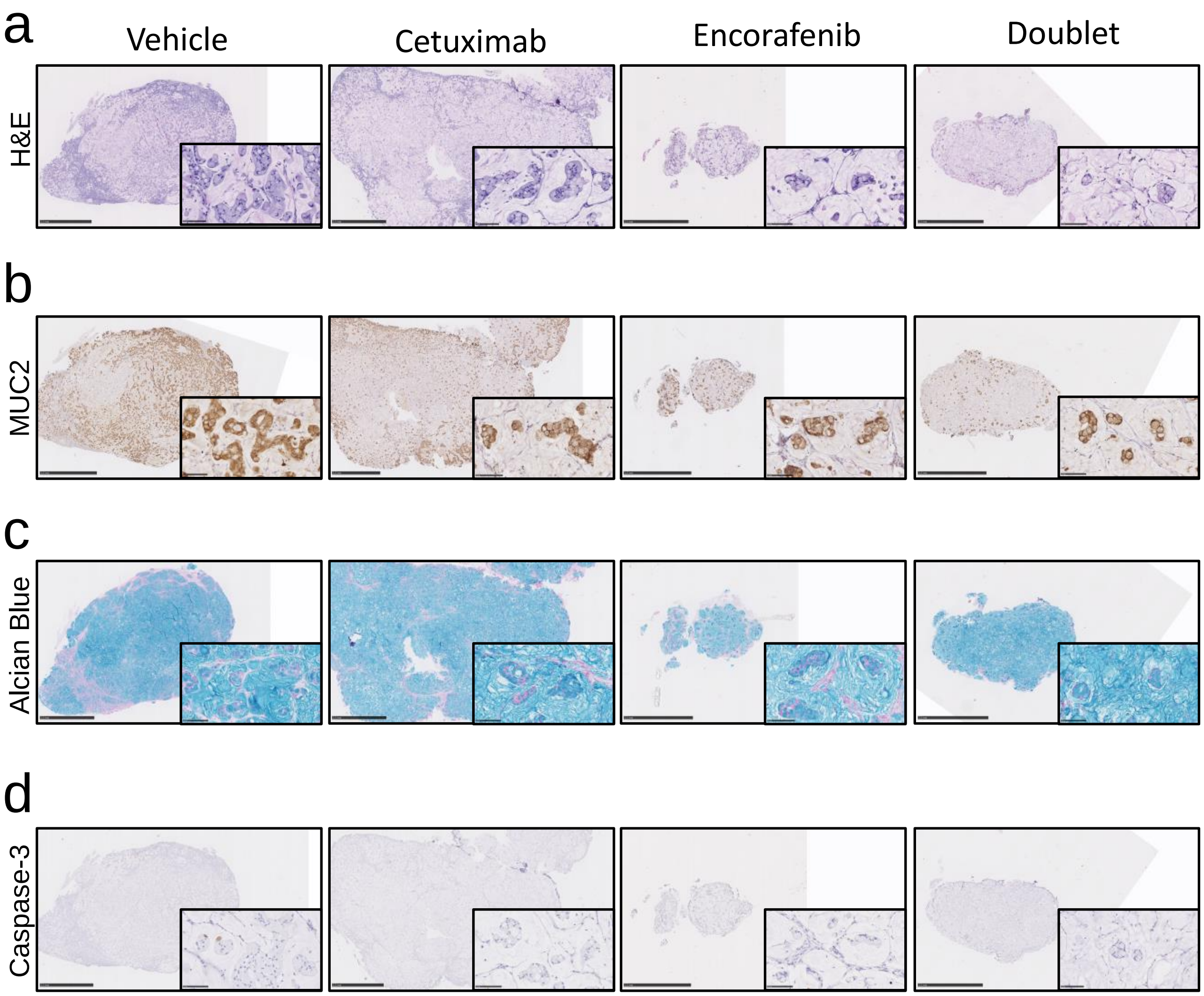

Supplementary Figure 8: Treatment with BRAF inhibitors reduces tumor cell proliferation in subcutaneous *BRAF*^{V600E} PMP-PDX tumors without impacting in apoptosis. a-d) Mice bearing subcutaneous *BRAF* mutant PMP-PDX tumors (PMP5.1) were treated with vehicle, cetuximab, encorafenib or doublet. At the end of the experiment, animals were euthanized, and tumors removed. Tumor sections and immunohistochemistry staining were performed from all samples. Images from a representative tumor from each group are presented for the following staining: Hematoxylin & Eosin(H&E) (**a**), MUC2 (**b**), Alcian blue (**c**) and caspase-3 (**d**). Scale bar 2.5 mm and 100 µm. PMP = Pseudomyxoma peritonei, PDX = Patient-derived xenografts.
