# Supplementary material for: Precision Oncology and Systemic Targeted Therapy in Pseudomyxoma Peritonei
Source: Clin Cancer Res. 2024 Jul 11;30(18):4082–99. doi: 10.1158/1078-0432.CCR-23-4072 (PMC11393541; doi:10.1158/1078-0432.CCR-23-4072)
Supplement: Supplementary Figure 9 — BRAF inhibitors reduce tumor mass in an orthotopic BRAFV600E PMP-PDX model. [file ccr-23-4072_supplementary_figure_9_suppsf9.pdf]

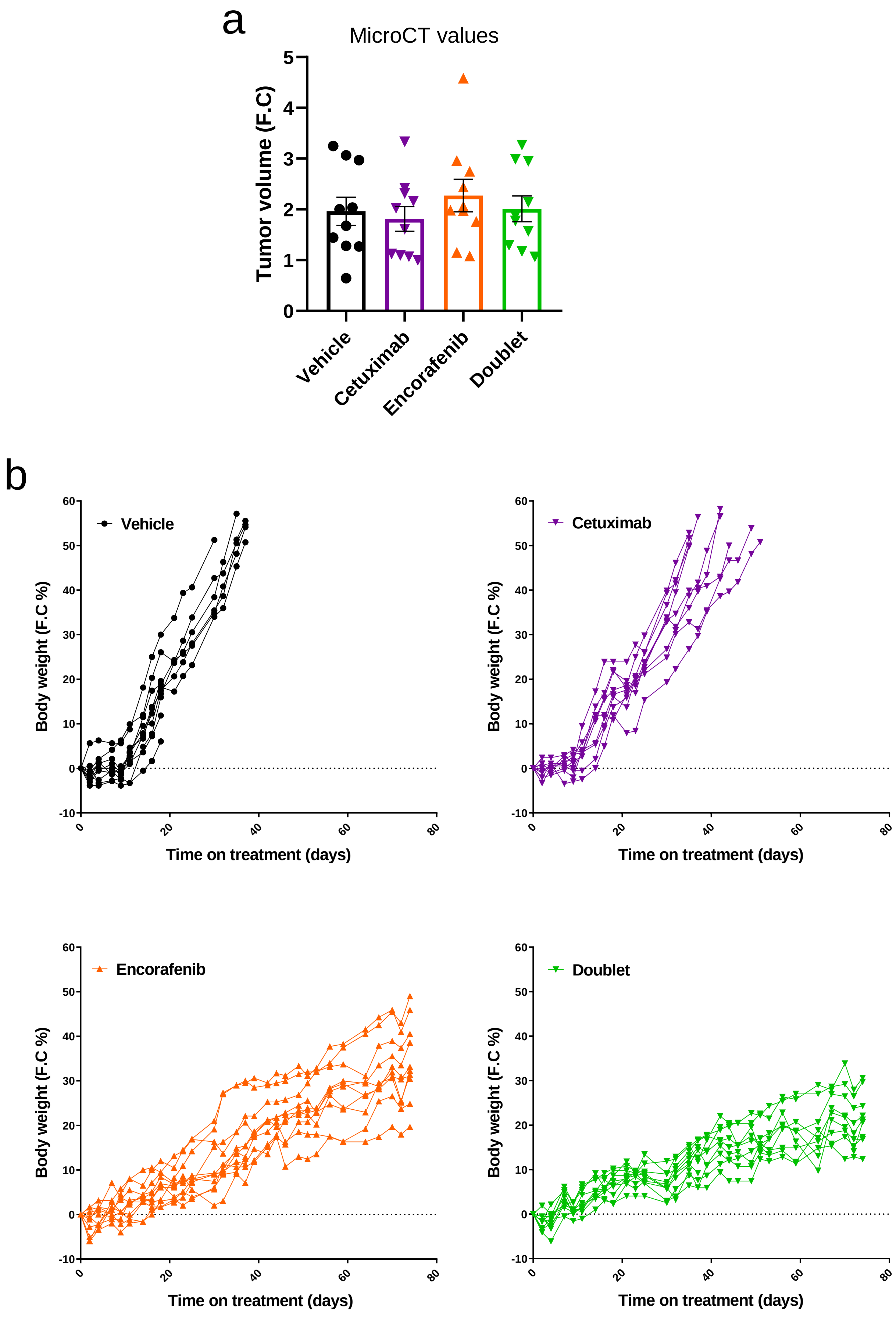
a
MicroCT values
Tumor volume (F.C)
0
1
2
3
4
5
Vehicle
Cetuximab
Encorafenib
Doublet
b
Vehicle
Cetuximab
Encorafenib
Doublet
Body weight (F.C %)
Time on treatment (days)
-10
0
10
20
30
40
50
60
0
20
40
60
80

Supplementary Figure 9: BRAF inhibitors reduce tumor mass in an orthotopic *BRAF*^{V600E} PMP-PDX model. Based on the microCT images, animals implanted with orthotopic PMP tumors (PMP5.1) were divided in four groups and treated with vehicle, cetuximab, encorafenib or doublet. **a**) Graph representing the fold change (F.C) tumor volume. Mean ± SEM is shown. Significant differences were assessed using one-way ANOVA and Tukey's multiple comparisons tests (*p value < 0.05, **p value < 0.01, ***p value < 0.001, ****p value < 0.0001). **b**) Representation of the fold change in percentage of body weight in each mouse of the treated groups overtime. PMP = Pseudomyxoma peritonei, PDX = Patient-derived xenografts.
