# Supplementary material for: Precision Oncology and Systemic Targeted Therapy in Pseudomyxoma Peritonei
Source: Clin Cancer Res. 2024 Jul 11;30(18):4082–99. doi: 10.1158/1078-0432.CCR-23-4072 (PMC11393541; doi:10.1158/1078-0432.CCR-23-4072)
Supplement: Supplementary Figure 10 — BRAF inhibitor treatment impacts in the mucinous structure of PMP tumors. [file ccr-23-4072_supplementary_figure_10_suppsf10.pdf]

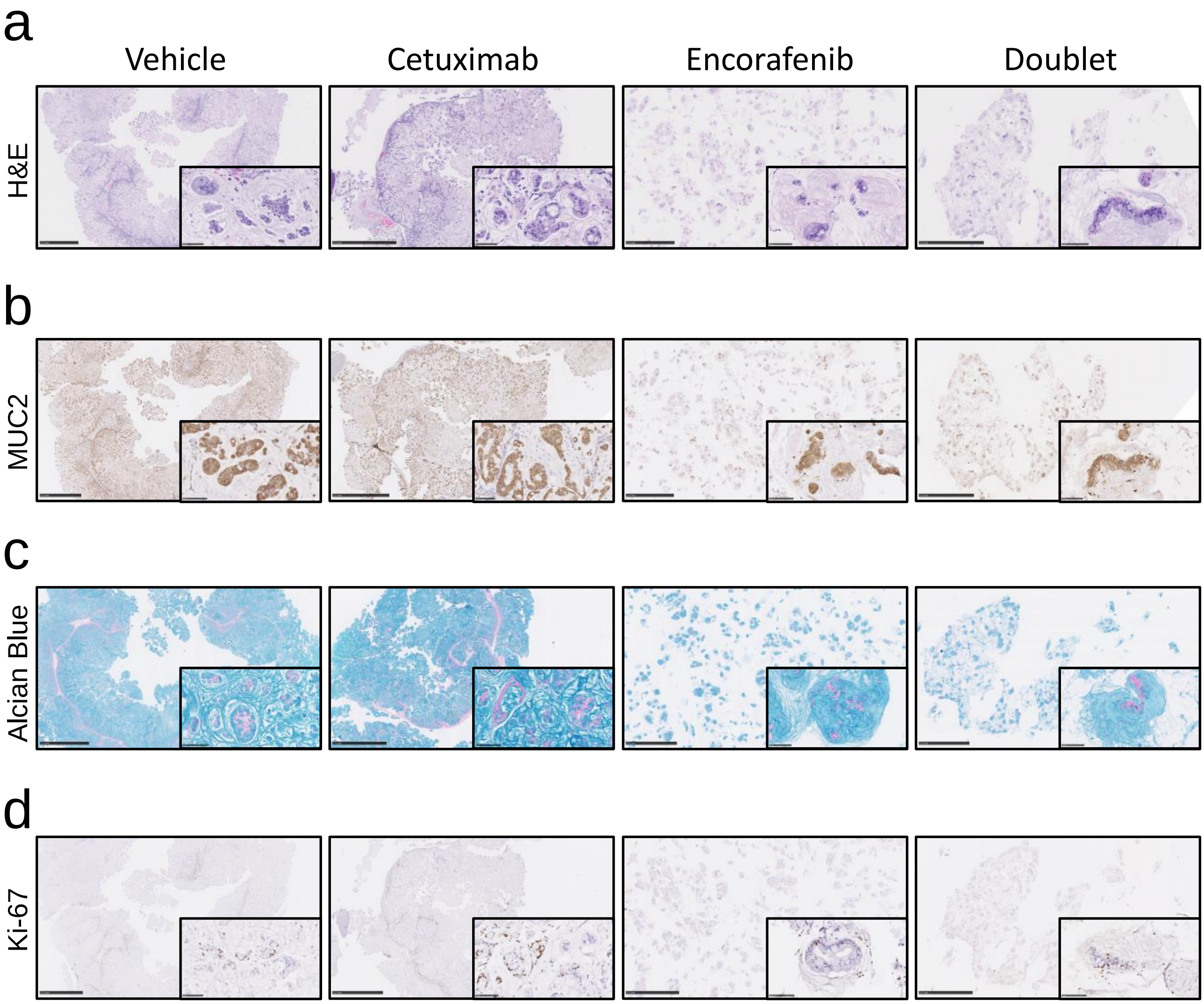

Supplementary Figure 10: BRAF inhibitor treatment impacts in the mucinous structure of PMP tumors. a-d) Mice bearing orthotopic *BRAF* mutant PMP-PDX tumors (PMP5.1) were treated with vehicle, cetuximab, encorafenib or doublet until they reached the endpoint criteria (vehicle and cetuximab) or until the end of the experiment (encorafenib and doublet). At this point, animals were euthanized, and mucinous tumor mass removed. Tumor sections and immunohistochemistry staining were performed from all samples. Images from a representative mouse from each group are presented for the following staining: Hematoxylin & Eosin (H&E) (**a**), MUC2 (**b**), Alcian blue (**c**), Ki-67 (**d**). Scale bar 2.5 mm and 100 µm. PMP = Pseudomyxoma peritonei, PDX = Patient-derived xenografts.
